# Transition Metal Dichalcogenides: Making Atomic‐Level Magnetism Tunable with Light at Room Temperature

**DOI:** 10.1002/advs.202304792

**Published:** 2023-12-10

**Authors:** Valery Ortiz Jimenez, Yen Thi Hai Pham, Da Zhou, Mingzu Liu, Florence Ann Nugera, Vijaysankar Kalappattil, Tatiana Eggers, Khang Hoang, Dinh Loc Duong, Mauricio Terrones, Humberto Rodriguez Gutiérrez, Manh‐Huong Phan

**Affiliations:** ^1^ Department of Physics University of South Florida Tampa FL 33620 USA; ^2^ Nanoscale Device Characterization Division National Institute of Standards and Technology Gaithersburg MD 20899 USA; ^3^ Department of Physics The Pennsylvania State University University Park PA 16802 USA; ^4^ Center for Computationally Assisted Science and Technology and Department of Physics North Dakota State University Fargo ND 58108 USA; ^5^ Department of Physics Montana State University Bozeman MT 59717 USA

**Keywords:** transition metal dichalcogenides, heterostructures, optospintronics, spin‐caloritronics, valleytronics

## Abstract

The capacity to manipulate magnetization in 2D dilute magnetic semiconductors (2D‐DMSs) using light, specifically in magnetically doped transition metal dichalcogenide (TMD) monolayers (*M*‐doped *TX*_2_, where *M* = V, Fe, and Cr; *T* = W, Mo; *X* = S, Se, and Te), may lead to innovative applications in spintronics, spin‐caloritronics, valleytronics, and quantum computation. This Perspective paper explores the mediation of magnetization by light under ambient conditions in 2D‐TMD DMSs and heterostructures. By combining magneto‐LC resonance (MLCR) experiments with density functional theory (DFT) calculations, we show that the magnetization can be enhanced using light in V‐doped TMD monolayers (e.g., V‐WS_2_, V‐WSe_2_). This phenomenon is attributed to excess holes in the conduction and valence bands, and carriers trapped in magnetic doping states, mediating the magnetization of the semiconducting layer. In 2D‐TMD heterostructures (VSe_2_/WS_2_, VSe_2_/MoS_2_), the significance of proximity, charge‐transfer, and confinement effects in amplifying light‐mediated magnetism is demonstrated. We attributed this to photon absorption at the TMD layer that generates electron–hole pairs mediating the magnetization of the heterostructure. These findings will encourage further research in the field of 2D magnetism and establish a novel design of 2D‐TMDs and heterostructures with optically tunable magnetic functionalities, paving the way for next‐generation magneto‐optic nanodevices.

## Graphical abstract

Both experimentally and theoretically it is demonstrated that the magnetization of a 2D dilute magnetic semiconductor based on a V‐doped transition metal dichalcogenide (TMD) monolayer or a 2D‐TMD heterostructure composed of metallic (magnetic) and semiconducting (non‐magnetic) TMD layers can be optically tuned. This establishes a novel direction for designing 2D‐TMDs and heterostructures with optically tunable magnetic functionalities for next‐generation nanodevices.

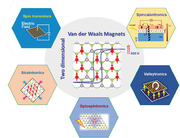

## 1 Introduction

Introducing spin to semiconductors has been a long sought‐after goal in search of supporting new semiconductor technologies beyond that of field effect transistors (FETs), leading to the proposition by Datta and Das of the spin field‐effect transistors (SFETs).^[^ ^1^, ^2^, ^3^, ^4^, ^5^, ^6^, ^7^ ^]^ In contrast to FETs that rely on electron charge, SFETs utilize electron spin and its alignment (up or down) to encode binary information, facilitating rapid information transmission with minimal power consumption.^[^ ^1^, ^4^ ^]^ Beyond SFETs, magnetic semiconductors present an exceptional platform for the development of a new generation of highly efficient spintronic devices, which would be compatible with existing semiconductor technologies. The ever‐increasing demand for denser electronics makes it crucial to reduce the dimensions of semiconductor materials.^[^ ^4^, ^7^, ^8^, ^9^ ^]^ As dimensions decrease, novel physical properties emerge, and new potential applications arise. However, miniaturization to the nanoscale might substantially impair their performance due to current leakage, rendering such devices inapplicable in ultrafast electronic nanodevices, particularly in supercomputers or future quantum computers.^[^ ^1^, ^7^ ^]^ For most magnetic semiconductors, ferromagnetic properties are largely weakened or even lost when their thickness is reduced to the atomic level or 2D limit.^[^ ^1^, ^2^, ^7^ ^]^

Recent advances in the domain of 2D van der Waals (vdW) magnetic materials have yielded unprecedented opportunities for exploiting atomically thin magnets and heterostructures with tunable magnetic, magnetoelectric, and magneto‐optical properties.^[^ ^10^, ^11^, ^12^, ^13^, ^14^, ^15^, ^16^, ^17^, ^18^, ^19^, ^20^, ^21^, ^22^, ^23^, ^24^, ^25^, ^26^, ^27^ ^]^ Among the identified 2D vdW magnets, the atomically thin intrinsic magnetic semiconductors CrI_3_ and Cr_2_Ge_2_Te_6_ have been extensively studied for their novel magnetoelectric, magneto‐optic, and spin transport properties.^[^ ^10^, ^11^, ^28^, ^29^, ^30^, ^31^, ^32^, ^33^, ^34^, ^35^, ^36^, ^37^ ^]^ Their magnetic functionalities can also be modulated by external stimuli (electric gating, strain, light).^[^ ^38^, ^39^, ^40^, ^41^, ^42^, ^43^, ^44^, ^45^, ^46^, ^47^ ^]^ Regrettably, these 2D semiconductors exhibit magnetic ordering at low temperatures (<50 K), limiting their practical implementation. Consequently, there is a growing demand for the development of 2D magnetic semiconductors that exhibit ferromagnetic ordering at ambient temperatures, under which most electronic devices operate.

**Figure 1:**
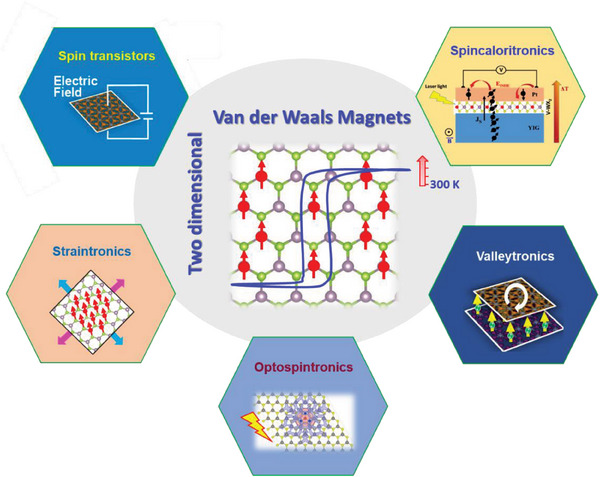
Perspectives of novel device applications of atomically thin magnetic transition metal dichalcogenides and their heterostructures.

2D transition metal dichalcogenides (2D‐TMDs) *TX*_2_ (*T* = W, Mo; *X* = S, Se, Te) are central to numerous vital device applications such as field‐effect transistors, photodetectors, photon emitters, valleytronics, and quantum computers.^[^ ^3^, ^4^, ^7^, ^8^, ^48^, ^49^ ^]^ With the exception of certain 2D‐TMDs such as VSe_2_,^[^ ^13^ ^]^ MnSe_2_,^[^ ^14^ ^]^ and CrSe_2_,^[^ ^50^ ^]^ which exhibit ferromagnetic ordering near room temperature but are *metallic*, the majority of *semiconducting* 2D‐TMDs including WSe_2_, WS_2_, and MoS_2_ monolayers are non‐magnetic or diamagnetic in nature.^[^ ^51^ ^]^ Recent studies have shown that introducing small quantities of magnetic transition metal atoms (e.g., V, Fe, Co, Cr, and Mn) can induce long‐range ferromagnetic order in these 2D‐TMDs at room temperature.^[^ ^52^, ^53^, ^54^, ^55^, ^56^, ^57^, ^58^, ^59^, ^60^, ^61^, ^62^, ^63^, ^64^, ^65^, ^66^, ^67^, ^68^, ^69^, ^70^, ^71^, ^72^, ^73^ ^]^ This approach presents a promising strategy to integrate extrinsic magnetic properties into atomically thin TMD semiconductors, giving rise to a novel class of 2D dilute magnetic semiconductors (2D‐DMSs). Due to their high‐quality interfaces and weakly coupled interlayer interactions, 2D‐TMDs with desirable properties can be easily stacked together, creating 2D vdW heterostructures with unique properties otherwise absent in their individual components.^[^ ^7^, ^47^, ^77^, ^78^, ^79^, ^80^, ^81^, ^82^, ^83^, ^84^, ^85^ ^]^ Their potential for next‐generation spintronic, opto‐spintronic, opto‐spin‐caloritronic, and valleytronic device applications has been emphasized, owing to their atomically thin nature and integrated opto‐electro‐magnetic properties.^[^ ^7^, ^82^, ^83^, ^84^, ^85^ ^]^ **Figure** **1** illustrates the potential applications of 2D‐TMD DMSs and their heterostructures.

The true appeal of 2D‐TMD DMSs for these applications stems from their magnetic tunability in response to external stimuli (electric gating, light, and strain). Magnetic state tunability of a 2D‐TMD can range from enhancing its magnetic moment, and tuning its Curie temperature to inducing magnetism in non‐magnetic materials through chemical doping,^[^ ^52^, ^53^, ^54^, ^55^, ^63^, ^86^, ^87^, ^88^ ^]^ defect engineering,^[^ ^60^ ^]^ phase change or structure engineering,^[^ ^75^, ^89^, ^90^ ^]^ interface engineering,^[^ ^47^, ^77^, ^78^, ^79^, ^80^, ^81^ ^]^ or applying external stimuli.^[^ ^56^, ^57^, ^91^ ^]^ Various strategies for enhancing magnetic functionalities in 2D‐TMDs have been highlighted in recent review articles.^[^ ^72^, ^92^ ^]^ Among these approaches, the capacity to modulate the magnetic moments of 2D‐TMDs reversibly, using external stimuli such as electric gating or light, appears to accommodate the ever‐increasing demands for multifunctional sensing devices, information storage, and quantum computing technologies. The recent discovery of tunable room temperature ferromagnetism in atomically thin V‐doped TMD (V‐WS_2_, V‐WSe_2_, V‐MoS_2_) semiconductors^[^ ^52^, ^53^, ^54^, ^61^ ^]^ has provided a new possibility for controlling their magnetic and magneto‐electronic properties through electrical and optical means.^[^ ^54^, ^56^, ^57^, ^59^, ^62^, ^93^, ^94^ ^]^ By combining the light‐tunable magnetism of 2D‐DMSs and the spin Seebeck effect,^[^ ^56^, ^94^, ^95^ ^]^ we have proposed an innovative strategy for the optic control of thermally driven spin currents across magnet/metal interfaces in spincaloritronic devices, potentially establishing a new subfield dubbed “Opto‐Spin‐Caloritronics”.^[^ ^83^ ^]^ To fully exploit the optically tunable magnetic properties of 2D‐TMD DMSs and their heterostructures for spintronics, spin‐caloritronics, straintronics, and valleytronics (Figure 1), it is essential to comprehend the underlying mechanisms of light‐mediated magnetism in these 2D systems.

**Figure 2:**
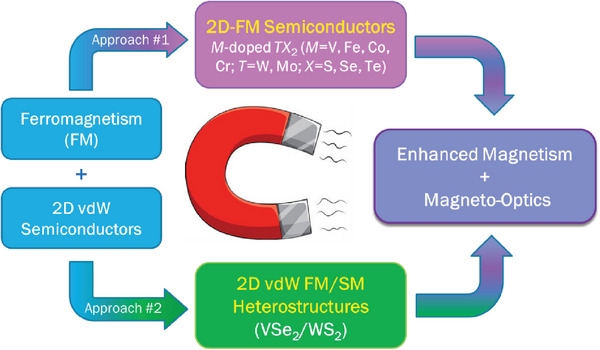
Approach 1: Introducing small amounts of magnetic atoms (e.g., V, Fe, Cr) into semiconducting van der Waals (vdW) TMD monolayers (e.g., WS_2_, WSe_2_, MoS_2_) creates a novel class of 2D vdW dilute magnetic semiconductors (e.g., V‐WS_2_, V‐WSe_2_, V‐MoS_2_). Approach 2: Interfacing 2D‐TMD magnets (e.g., VSe_2_, VS_2_, MnSe_2_, CrSe_2_) with 2D‐TMD semiconductors (e.g., WS_2_, WSe_2_, MoS_2_) create a novel class of magneto‐optic 2D vdW heterostructures (e.g., VSe_2_/WS_2_, VS_2_/MoS_2_, MnSe_2_/WSe_2_, CrSe_2_/WSe_2_). FM = Ferromagnetism; SM = Semiconductor.

In this Perspective article, we demonstrate how light modulates the magnetization in 2D‐TMD DMSs (V‐WS_2_, V‐WSe_2_, V‐MoS_2_) and associated heterostructures (VSe_2_/MoS_2_, VSe_2_/WS_2_), using an ultrasensitive magneto‐LC resonance (MLCR) magnetometer. Supplemented by DFT calculations, these findings enable us to propose innovative design strategies for novel 2D‐TMD DMSs and heterostructures with enhanced light‐tunable magnetic functionalities suitable for modern nanodevice applications. **Figure** **2** presents two promising approaches for creating such 2D‐TMDs and heterostructures, with their optically tunable magnetic properties discussed herein.

The paper is structured as follows: we first introduce the MLCR magnetometry technique and then present the results obtained using this technique to demonstrate the light‐mediated magnetism effects in the 2D‐TMD DMSs and related heterostructures. We will also discuss emerging opportunities and challenges in the field of study.

## 2 Magneto‐LC Resonance Magnetometry for Probing Light‐Mediated Magnetism

As ferromagnetic signals become exceedingly weak in atomically thinned magnetic systems, probing small changes in magnetization of the material subject to external stimuli, such as light, presents a considerable challenge.^[^ ^39^, ^41^, ^43^, ^96^, ^97^, ^98^, ^99^, ^100^, ^101^, ^102^, ^103^, ^104^, ^105^, ^106^, ^107^ ^]^ Superconducting quantum interference devices (SQUID) can measure the magnetization of 2D materials,^[^ ^52^, ^53^ ^]^ but they are not well‐suited for real‐time measurements while simultaneously illuminating the samples with light.^[^ ^97^ ^]^ Transport measurements on 2D materials also pose difficulties, as the size of the electrical contacts is often larger compared to the surface area of the sample.^[^ ^7^ ^]^ 1D edge contacts may be used instead, but obtaining reliable low‐resistance ohmic contacts on TMDs remains a challenge.^[^ ^108^, ^109^ ^]^ Optical methods based on the magneto‐optic Kerr effect (MOKE), time‐resolved Faraday rotation, and reflectance magneto‐circular dichroism (RMCD) have been successfully employed to characterize the light‐mediated magnetic properties of 2D materials such as Fe_3_GeTe_2_,^[^ ^96^ ^]^ Cr_2_Ge_2_Te_6_,^[^ ^40^ ^]^ and CrI_3_.^[^ ^39^ ^]^ However, the use of high laser powers in these methods may cause local heating and consequently thermal instability – a significant source of noise. The limitations of these techniques necessitate the development of a new approach to measure 2D magnetization in real time as external stimuli, such as light, are applied.

To probe the light‐induced magnetization of an atomically thin magnetic film, we have developed a novel magneto‐LC resonance (MLCR) magnetometer with ultrahigh magnetic field sensitivity (pT regime).^[^ ^56^, ^94^ ^]^

**Figure 3:**
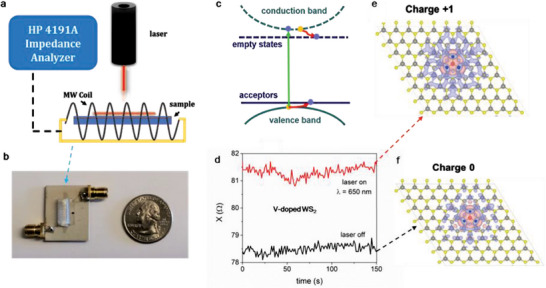
a) Schematic of the MLCR measurement setup. Changes in the coil's reactance corresponding to changes in the magnetic permeability or magnetization of a magnetic sample due to light irradiation are monitored in real‐time by a soft magnetic coil. The coil is mounted on a test fixture composed of a copper‐clad dielectric material, with its two ends soldered into the inner pin of SMA ports, as depicted in (b). c) Schematic of the photon‐induced magnetism effect when light illuminates a 2D‐TMD; d) Reactance as a function of time when the laser is off and on; The projected magnetic moment along the c‐axis of the V‐WS_2_ monolayer upon a single hole injection (e) relative to no charge injection (f). d) Both MLCR experiments and e,f,) DFT calculations confirm that the magnetization of the V‐WS_2_ monolayer increases upon light illumination.

**Figure** **3**a presents a schematic of the light‐mediated magnetization measurement system using the principle of MLCR.^[^ ^110^, ^111^ ^]^ The MLCR design draws inspiration from conventional magneto‐inductive coils and the sensitivity of the giant magneto‐impedance (GMI) effect,^[^ ^112^ ^]^ which has proven useful for detecting ultra‐small magnetic fields in biosensing applications and for structural health monitoring.^[^ ^113^, ^114^ ^]^ The sensor is constructed from a Co‐rich soft magnetic microwire exhibiting very high GMI ratios and magnetic field sensitivity. The melt‐extracted amorphous microwire, with a nominal composition of Co_69.25_Fe_4.25_Si_13_B_12.5_Nb_1_ and diameter of approximately 60 µm, is wound into a 15‐turn, 10‐mm‐long coil with a 5 mm internal diameter. The coil is mounted on a test fixture made of a copper‐clad dielectric material (Figure 3b). The two ends of the coil are soldered onto the inner pin of SMA ports, which are connected to a coaxial cable and terminated with a 50‐Ω cap. The coil is then driven by a frequency in the MHz range (≈118 MHz, near the coil's LC resonance, hence the name MLCR), and the impedance (*Z*), resistance (*R*), and reactance (*X*) are measured using an HP 4191A impedance analyzer.

The operating principle of the magnetic microwire coil (MMC) can be described using lumped element circuit theory. A simple model for a coil sensor is a lumped element representation of a non‐ideal inductor. Winding cylindrical conductors close to each other introduces parasitic elements *R*_par_ and *C*_par_, such that the non‐ideal inductor can be represented as a series combination of an ideal inductor *L* and *R*_par_, in parallel with *C*_par_. The impedance of the coil *Z_coil_* can then be written as

(1) $$Z_{\mathrm{coil}}=Z_{R_{\mathrm{par}}}+Z_{C_{\mathrm{par}}}$$

(2) $$Z_{\mathrm{coil}}=\frac{1}{\frac{1}{R_{\mathrm{par}}+j\omega L}+\frac{1}{-j/\omega C_{\mathrm{par}}}}$$

(3) $$Z_{\mathrm{coil}}=\frac{R_{\mathrm{par}}+j\omega \left[L\left(1-\omega^{2}LC_{\mathrm{par}}\right)-C_{\mathrm{par}}R_{\mathrm{par}}^{2}\right]}{{\left(1-\omega^{2}LC_{\mathrm{par}}\right)}^{2}+{\left(\omega C_{\mathrm{par}}R_{\mathrm{par}}\right)}^{2}}$$

where ω is the angular frequency, and *j* is the imaginary unit. Resonance occurs when the inductive reactance (*X*_L_) and the capacitive reactance (*X*_C_) have equal magnitudes but differ in phase by 180 degrees. In this case, minimal current flows through the wire, the impedance of the coil becomes very large, and self‐resonance is achieved. This resonance frequency is given by:

(4) $$f_{0}=\frac{\sqrt{1-\left(R_{\mathrm{par}}^{2}C_{\mathrm{par}}/L\right)}}{2\pi \sqrt{LC_{\mathrm{par}}}}$$

The reactance of the coil is of particular interest to utilize the coil for detecting changes in the magnetic permeability within its core. The impedance of the coil has the general form:

(5) $$Z_{\mathrm{coil}}=R+jX$$

Therefore, we can extract the reactance from the imaginary component of Equation (3):

(6) $$X_{\mathrm{coil}}=\frac{\omega \left[L\left(1-\omega^{2}{\mathrm{LC}}_{par}\right)-C_{\mathrm{par}}R_{par}^{2}\right]}{{\left(1-\omega^{2}LC_{par}\right)}^{2}+{\left(\omega C_{par}R_{par}\right)}^{2}}$$

To measure the magnetic permeability or magnetization of a magnetic thin film, such as a 2D‐TMD DMS, subjected to an external stimulus such as light, the magnetic film is positioned within the core of the coil, and the reactance of the coil is measured during light irradiation. According to Equation (6), the reactance (*X*) of the coil is strongly dependent on the induction (*L*) through the coil's core. Since the film is ferromagnetic, it will alter the relative permeability of the space within the coil, thereby changing the magnetic flux through the coil and consequently the reactance of the coil. As the microwire itself is ferromagnetic, the magnetization of the film will also lead to a change in the effective permeability of the microwire. Thus, the reactance of the sensor depends on this effective permeability, *X* = *X*(µ_eff_). Changes in the permeability of the film upon light illumination (i.e., the presence of additional holes mediating the magnetism in 2D‐TMDs) will influence the effective permeability of the coil, which can be accessed through the change in its reactance: Δ*X* = *X*(µ_eff_, *laser* *on*) − *X*(µ_eff_, *laser* *off*). This change in reactance (Δ*X*) is proportional to the change in magnetization Δ*M* of the film upon light illumination, as illustrated in Figure 3c–f for the case of a V‐doped WS_2_ monolayer. Using the MLCR method, we investigate the optically tunable magnetic properties of selected 2D‐TMD DMSs and their heterostructures, with some of the results presented and discussed below.

While this method proved successful in characterizing these 2D‐TMD DMSs, it is not without its limitations. Commercial availability of magnetic microwires with a suitable composition that exhibits the ultra‐high sensitivity we reported is limited. Alternatives may be available, but each MMC sensor requires carful characterization, even when made with wires with the same nominal compositions. While MMC sensors operate with high sensitivity at room temperature, their temperature dependence is yet to be studied. Another limitation of this measurement is the difficulty to calibrate Δ*X* to a specific value of Δ*M* since each individual sample will modify the coil's transfer function due to the microwire's magnetic properties. In summary, the MLCR technique provides a new tool to characterize 2D magnetic materials and is an excellent complement to other established magnetometry.

## 3 Light‐Tunable 2D Magnetism

### 3.1 Magnetically Doped Transition Metal Dichalcogenide Monolayers

**Figure 4:**
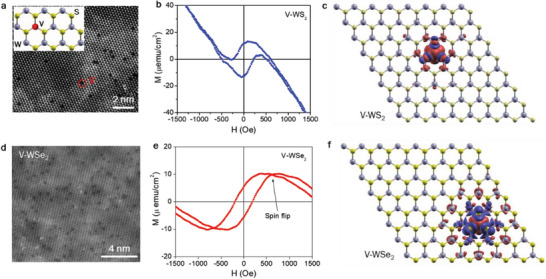
HRTEM images, magnetic hysteresis loops *M*(*H*), and spin configurations of a,b,c) 2 at.% V‐doped WS_2_ and d,e,f) 4 at.% V‐doped WSe_2_ monolayers, respectively. Within the V‐WS_2_ monolayer, the magnetic moment of V (replacing one W) is *ferromagnetically* coupled to the magnetic moments induced at the neighboring W sites (all red, see panel c). In the V‐WSe_2_ monolayer, the magnetic moment of V is *antiferromagnetically* coupled to the magnetic moments at the nearest W sites (blue vs red, see panel f) but *ferromagnetically* coupled to those at the further distance W sites (red, see panel f). It is the antiferromagnetic (AFM) coupling between the V and W spins at the nearest distance that leads to the thermally induced spin‐flipping phenomenon manifested as a crossover of magnetization in the *M*(*H*) loop. Panels (a,b) are reproduced with permission.^[^ ^52^ ^]^ Copyright 2020, Wiley‐VCH; Panels (d,e) are reproduced with permission.^[^ ^53^ ^]^ Copyright 2020, Wiley‐VCH.

Among 2D vdW materials, 2D‐TMDs are a fertile ground for novel quantum phenomena including nontrivial electronic topology, non‐saturating giant magnetoresistance, and topological field‐effect transistors.^[^ ^1^, ^3^, ^4^, ^7^ ^]^ Recently, TMD monolayers (e.g., WS_2_, WSe_2_, and MoS_2_) doped with magnetic transition metal atoms (e.g., V, Fe, Co, and Cr) have been reported to exhibit room‐temperature ferromagnetic order, emerging as a novel class of 2D‐DMSs.^[^ ^52^, ^53^, ^54^, ^55^, ^56^, ^57^, ^58^, ^59^, ^60^, ^61^, ^62^, ^63^, ^64^, ^65^, ^66^, ^67^, ^68^, ^69^, ^70^, ^71^ ^]^ We have discovered tunable room‐temperature ferromagnetism in V‐WS_2_ and V‐WSe_2_ monolayers by varying the V‐doping concentration.^[^ ^52^, ^53^ ^]^ The origin of long‐range magnetic order in 2D DMSs has been previously studied by multiple groups.^[^ ^115^, ^116^, ^117^ ^]^ Two possible mechanisms have been proposed: i) direct exchange interactions between magnetic dopants which are shortly spaced, and ii) an indirect exchange interaction mediated by itinerant carriers, also known as the Ruderman–Kittel–Kasuya‐Yosida (RKKY) or the Zerner model. In the V‐WS_2_ and V‐WSe_2_ monolayers we present, the V‐doping concentration is low (≈1–4 at.%) and the spacing between dopants is large, leading us to conclude that the RKKY interaction is mainly responsible for the observed magnetic ordering. This is in line with the work of Song et al. that shows evidence of the long‐range magnetic order in the V‐WSe_2_ monolayer due to the presence of itinerant spin‐polarized holes that mediate ferromagnetic interactions between the magnetic moments of V‐dopants.^[^ ^116^ ^]^ In TMDs, the spin–orbit coupling from the heavy transition metals also plays a significant role in the spin polarization of the carriers near their sites, further contributing to the magnetic ordering introduced by the dopants.^[^ ^115^ ^]^ **Figure** **4** highlights some notable features of these 2D magnets.

By varying V‐doping concentrations, we have demonstrated an enhanced magnetization and achieved the highest doping levels ever attained for atomically thin vanadium‐doped TMDs (≈2 at.% for V‐doped WS_2_ monolayers,^[^ ^52^ ^]^ and ≈4 at.% for V‐doped WSe_2_ monolayers).^[^ ^53^ ^]^ Unlike the case of V‐WS_2_ monolayers (Figure 4b,c), we have observed the thermally induced spin flipping (TISF) phenomenon in V‐WSe_2_ monolayers (Figure 4e) due to the presence of antiferromagnetic coupling between spins at V‐sites and their nearest W sites (Figure 4f). Interestingly, the TISF phenomenon can be achieved at low magnetic fields (less than 100 mT) and manipulated by modifying the vanadium concentration within the WSe_2_ monolayer. These 2D DMSs can thus be used as novel 2D spin filters to enhance the spin‐to‐charge conversion efficiency of spin‐caloritronic devices.^[^ ^83^, ^118^ ^]^ It has been reported that after magnetic transition metal (e.g., V or Fe) doping, the photoluminescence signal is strongly suppressed in 2D‐TMDs,^[^ ^52^, ^53^, ^55^, ^61^ ^]^ which has been attributed to the formation of impurity energy bands caused by p‐type doping at the valence band maximum.^[^ ^51^, ^52^, ^53^, ^72^ ^]^ Therefore, it is crucial to select appropriate doping concentrations at which 2D‐TMDs exhibit optimized magnetic and optical properties.

Duong et al. studied the effect of electric gating on the magnetization of V‐doped WSe_2_ monolayers and found that hole injection enhances the magnetization, while electron injection significantly decreases it.^[^ ^54^, ^57^ ^]^ Complementing experimental findings, DFT calculations reveal the dominant hole‐mediated long‐range ferromagnetic interactions between V‐spins in atomically thin V‐doped TMD systems.^[^ ^57^ ^]^ Our previous studies have shown that, after vanadium doping, significant photoluminescence is still present in 2 at.% V‐doped WS_2_ and 4 at.% V‐doped WSe_2_ monolayers,^[^ ^52^, ^53^ ^]^ which both exhibit the largest saturation magnetization (*M_S_*) values among the compositions investigated. These observations have led us to propose that the ferromagnetism in the V‐WS_2_ or V‐WSe_2_ monolayer can be mediated by illumination with a laser of appropriate energy, specifically, above the optical gap (Figure 3c). Electrons from photogenerated electron–hole pairs may be captured by the V atoms, thus creating an imbalance in the carrier population (i.e., the generation of excess holes) such that the ferromagnetism of the monolayer is modified (Figure 3d–f).

**Figure 5:**
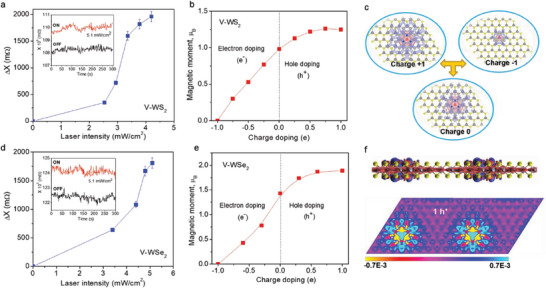
The laser intensity dependence of the change in reactance (Δ*X*) of a) V‐WS_2_ and d) V‐WSe_2_ monolayers. Insets show the change in reactance upon light illumination with a 650 nm laser for a) V‐WS_2_ and d) V‐WSe_2_ monolayers; The net magnetic moments of b) V‐WS_2_ and e) V‐WSe_2_ monolayers with different carrier doping densities; c) The projected magnetic moment along the c‐axis of the V‐WS_2_ monolayer upon one hole and one electron injection relative to no charge injection; f) The projected magnetic moment along the c‐axis of the V‐WSe_2_ monolayer upon a single hole injection. Hole injection increases the magnetization of the V‐WS_2_ or V‐WSe_2_ monolayer. Panels (a,b,c) are produced with permission.^[^ ^56^ ^]^ Copyright 2021, Wiley‐VCH; Panel (f) are produced with permission.^[^ ^57^ ^]^ Copyright 2020, APS.

The combination of magnetic and semiconducting properties in 2D‐TMD DMSs has indeed enabled light modulation and tunability of magnetization, as demonstrated for V‐WS_2_ and V‐WSe_2_ monolayers (**Figure** **5**). As can be clearly seen from Figure 5a,d, both V‐WS_2_ and V‐WSe_2_ systems exhibit a similar light intensity‐dependent magnetization trend. Note that undoped TMD samples (pristine WS_2_ and WSe_2_ monolayers) do not exhibit light‐mediated magnetism, and the observed enhancement of the magnetization in illuminated V‐doped TMD monolayers is not due to a laser/sample heating effect but originates from carrier‐mediated ferromagnetism, similar to the case of a p‐type (In,Mn)As/GaSb semiconductor.^[^ ^119^ ^]^ DFT calculations demonstrate that hole injection shifts the Fermi level deeper inside the valence band, while electron injection shifts it toward the conduction band edge.^[^ ^56^, ^57^ ^]^ As a result, the magnetic moment becomes larger with increasing hole concentration, while an opposite trend is observed for electron injection (Figure 5b,e). Increasing the concentration of holes results in a more robust magnetic moment across the lattice, where W atoms far from the V site exhibit an enhanced magnetic moment. Since long‐range ferromagnetic interactions are mediated by free holes in V‐WS_2_ and V‐WSe_2_ systems it is unsurprising that optically injecting hole‐carriers leads to enhanced ferromagnetism. The theoretical calculations fully support the experimental findings. It is worth noting that at large hole concentrations, the magnetic moment saturates, confirming the feature observed experimentally (Figure 5a,d). This has been attributed to the screening of charge carriers at high hole concentrations. The experimental results presented in Figure 5 were obtained from MLCR experiments conducted using a 650 nm laser. We also performed MLCR measurements on the same samples using a 520 nm laser and observed enhanced magnetization, confirming that light‐enhanced magnetization can be achieved with any wavelength above the optical gap.

Although the V‐WS_2_ and V‐WSe_2_ systems share a similar light‐mediated magnetization effect (Figure 5a,d), a noticeable difference in magnetic coupling between spins at a V‐site and its nearest W‐sites (namely, the nearest V‐W spins) is evident.^[^ ^56^, ^57^ ^]^ DFT calculations reveal that interactions between the nearest V‐W spins are *ferromagnetic* in V‐WS_2_ monolayers but *antiferromagnetic* in V‐WSe_2_ monolayers at the same V‐doping level.^[^ ^52^, ^53^, ^56^, ^57^ ^]^ In the case of V‐WS_2_ monolayers, the ferromagnetic interaction between the nearest V‐W spins becomes stronger with increasing hole concentration (or increase of light intensity), giving rise to an enhanced magnetic moment (Figure 5c). However, the situation is rather different for V‐WSe_2_ monolayers (Figure 5f), in which the V atom couples antiferromagnetically to the nearest W sites, and ferromagnetically to the distant W‐sites. The introduction of charge carriers mediates this interaction, where increasing hole carriers results in an enhanced magnetic moment at the V site. Additionally, the magnetic moment at the near W sites, flips from antiferromagnetic to weakly ferromagnetic, which combined with the increasing magnetic moment at the V site, results in the enhanced long‐range ferromagnetism (Figure 5f). What is particularly striking in the V‐WSe_2_ system, is the antiferromagnetic coupling between V and near W sites, which appears to be responsible for the lack of saturation of the magnetic moment at higher laser intensities (Figure 5d).

These findings suggest that the light‐mediated magnetism effect is *universal* to the class of 2D‐TMD DMS and should be fully exploited for applications in opto‐spintronics, opto‐spin‐caloritronics, spin‐valleytronics, and quantum communications.

### 3.2 2D Transition Metal Dichalcogenide Heterostructures

Typical vdW TMD monolayers offer extensive flexibility and integration with one another.^[^ ^7^, ^8^, ^15^, ^17^, ^51^ ^]^ Stacking different 2D‐TMDs can create novel heterostructures with atomically sharp interfaces and properties that would otherwise be absent in their individual components.^[^ ^15^, ^17^, ^51^ ^]^ Recent studies have demonstrated that the magnetic or magneto‐optical properties of a non‐magnetic TMD (e.g., WS_2_, MoS_2_) can be induced or enhanced by stacking it with another magnetic TMD (VS_2_, VSe_2_, CrSe_2_, MnSe_2_).^[^ ^13^, ^50^, ^120^, ^121^, ^122^ ^]^ This occurs as a result of combined charge transfer and magnetic proximity (PM) effects.^[^ ^13^, ^50^, ^79^, ^122^ ^]^ By forming MoS_2_/VS_2_ and WS_2_/VS_2_ interfaces, DFT calculations indicate that charge transfer occurs across the interface from MoS_2_ or WS_2_ to VS_2_, as both MoS_2_ and WS_2_ have smaller work functions compared to VS_2._^[^ ^122^ ^]^ Electrons accumulate in the VS_2_ layer, while holes occupy the MoS_2_ or WS_2_ layer. Consequently, both MoS_2_/VS_2_ and WS_2_/VS_2_ heterostructures exhibit enhanced magnetic properties, with Curie temperatures exceeding 300 K.^[^ ^122^ ^]^ By forming CrSe_2_/WSe_2_ interfaces, DFT calculations by Li et al. also show that charge transfer from the WSe_2_ to the CrSe_2_ layer and interlayer coupling within CrSe_2_ play crucial roles in the magnetic properties of the heterostructure.^[^ ^50^ ^]^ A similar situation is anticipated in VSe_2_/MoS_2_ and MnSe_2_/MoTe_2_ systems,^[^ ^13^, ^121^ ^]^ where both VSe_2_ and MnSe_2_ monolayers have been reported to display ferromagnetism above room temperature.^[^ ^13^, ^14^ ^]^ The combined semiconducting and ferromagnetic properties make these heterostructures appealing for opto‐spintronics and opto‐spin‐caloritronics, as the application of external stimuli such as electric gating and light is likely to promote the charge transfer process and hence alter the magnetic and magneto‐optic properties of the heterostructures.^[^ ^89^ ^]^

As demonstrated above for V‐WS_2_ and V‐WSe_2_ monolayers, the magnetic and semiconducting properties coexist, giving rise to their magneto‐optical properties and granting access to the rich electronic properties of 2D semiconductors, enabling the light tunability of magnetization. These findings suggest a similar phenomenon in VSe_2_/WS_2_ and VSe_2_/MoS_2_ heterostructures. An intriguing feature of these heterostructures is the presence of strong interfacial magnetic coupling and the potential for charge transfer between the two layers, which may play a key role in mediating the magnetization of the film. Bilayer MoS_2_ shows a strong photon absorption peak ≈600–700 nm, and by illuminating the VSe_2_/MoS_2_ film with energy close to the peak, we expect considerable photogeneration of electron–hole pairs. These pairs may be separated through the electric field at the heterointerface, which could lead to light‐tunable magnetism in the film.

**Figure 6:**
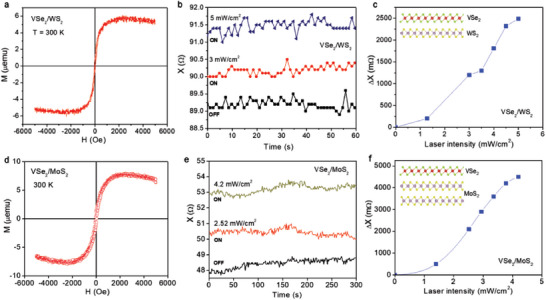
Magnetic hysteresis loops (*M*(*H*)) taken at 300 K for a) 1L‐VSe_2_/1L‐WS_2_ and d) 1L‐VSe_2_/2L‐MoS_2_ films. The reactance (*X*) versus time upon illumination with a 650‐nm laser with various intensities for b) 1L‐VSe_2_/1L‐WS_2_ and e) 1L‐VSe_2_/2L‐MoS_2_ films. Laser intensity dependence of the reactance change (Δ*X*) for c) 1L‐VSe_2_/1L‐WS_2_ and f) 1L‐VSe_2_/2L‐MoS_2_ films.

To investigate this hypothesis, we conducted MLCR experiments on both VSe_2_/WS_2_ and VSe_2_/MoS_2_ heterostructure samples upon light illumination using a diode laser with a wavelength of ≈650 nm (*hν* ≈1.91 eV). In this case, the VSe_2_/WS_2_ or VSe_2_/MoS_2_ heterostructure consists of a vertically stacked monolayer (1L) VSe_2_ and monolayer (1L) WS_2_ or bilayer (2L) MoS_2_ grown on an SiO_2_ substrate by combining molecular beam epitaxy (MBE) and chemical vapor deposition (CVD), respectively. Representative results of the VSe_2_/WS_2_ and VSe_2_/MoS_2_ samples are displayed in **Figure** **6**. It is noteworthy to observe that both the 1L‐VSe_2_/1L‐WS_2_ and 1L‐VSe_2_/2L‐MoS_2_ samples exhibit a pronounced ferromagnetic signal at room temperature (Figure 6a,d), as well as light‐tunable ferromagnetism at room temperature (Figure 6b,e). For both heterostructures, the magnetization significantly increases with increasing laser intensity and tends to saturate at high laser intensities (Figure 6c,f). The light intensity dependence of magnetization for the VSe_2_/WS_2_ and VSe_2_/MoS_2_ heterostructures (Figure 6c,f) is similar to that observed for V‐doped TMD monolayers (Figure 5a,d) and for a CH_3_NH_3_PbI_3_/LSMO heterostructure.^[^ ^100^ ^]^ The enhancement and tunability of light‐mediated ferromagnetism in the 1L‐VSe_2_/2L‐MoS_2_ film were also independently confirmed by the pump‐probe MOKE technique.^[^ ^94^ ^]^ It is important to note that this light‐mediated magnetism effect is not observed in the individual layers (WS_2_, MoS_2_, VSe_2_); only when they are stacked together do we observe light‐dependent magnetization. These results demonstrate the universality of the light‐mediated magnetism effect and pave a new pathway for the design and fabrication of novel van der Waals heterostructures for use in 2D van der Waals spintronics, opto‐spin‐caloritronics, and opto‐valleytronics.

**Figure 7:**
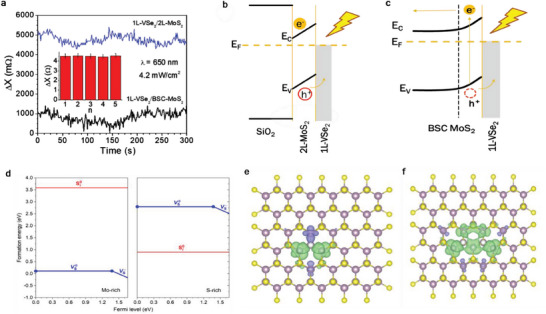
a) Comparison of the reactance change (Δ*X*) due to light irradiation at the same wavelength (*λ* = 650 nm) and intensity (4.2 mW cm^−2^) between the 1L‐VSe_2_/2L‐MoS_2_ film and the 1L‐VSe_2_/BSC‐MoS_2_ film. Upon light irradiation, the enhancement of the magnetization is approximately 4 times greater in the 1L‐VSe_2_/2L‐MoS_2_ film than in the 1L‐VSe_2_/BSC‐MoS_2_ film. Inset of a) shows values of the reactance change (Δ*X*) measured at different times demonstrating the reproducibility of the observed effect. Schematics show a possible charge transfer via b) the 2L‐MoS_2_/VSe_2_ and c) BSC‐MoS_2_/VSe_2_ interface upon light irradiation; d) Formation energies of sulfur vacancies (*V*_S_) and interstitials (S*_i_*) in a 2*H*‐MoS_2_ bilayer under extreme Mo‐rich and S‐rich conditions and plotted as a function of the Fermi level from the VBM to the CBM. The slope of energy‐line segments indicates the charge state. The vacancy (interstitial) is found to be the dominant native point defect under the Mo‐rich (S‐rich) condition. *V*_S_ is stable as the neutral (and nonmagnetic) vacancy *V*_S_^0^ in a wide range of Fermi‐level values and as the negatively charged vacancy *V*_S_^–^ near the CBM (i.e., under n‐type conditions). The (0/–) transition level of *V*_S_ is at 0.31 eV below the CBM. *V*_S_^–^ has a calculated magnetic moment of 1*µ*_B_. S*_i_* is, on the other hand, stable as the neutral and nonmagnetic vacancy S*_i_* ^0^ in the entire range of Fermi‐level values; e) Top‐view of spin density associated with a single negatively charged defect *V*_S_^–^; (f) top‐view of the spin density associated with a pair of *V*_S_^–^. Large (purple) spheres are Mo, and small (yellow) spheres are S. The lattice site of the vacancy is marked by a small (white) sphere. The isosurface level is set to 0.014 e/Å^3^ and the green (purple) isosurfaces correspond to up (down) spin.

Comparison of the light‐mediated magnetization results of 1L‐VSe_2_/2L/MoS_2_ and 1L‐VSe_2_/BSC MoS_2_ (BSC: Bulk single crystal) samples has shown that the change in magnetization in 1L‐VSe_2_/2L‐MoS_2_ is approximately 4 times greater than that of 1L‐VSe_2_/BSC‐MoS_2_ (**Figure** **7**a) and this result is reproducible (inset of Figure 7a). This observation leads us to believe that electron confinement effects on the 2L‐MoS_2_ might play a significant role in the mechanism behind light mediated magnetism in this heterostructure (Figure 7b), as compared to the case of BSC MoS_2_ (Figure 7c).

To elucidate the mechanism of light‐enhanced magnetism in the 1L‐VSe_2_/2L‐MoS_2_ system, we conducted first‐principles defect calculations based on the Heyd‐Scuseria‐Ernzerhof (HSE) hybrid functional (with the mixing parameter set to 0.15),^[^ ^123^, ^124^ ^]^ as implemented in VASP with the van de Waals correction and finite‐supercell size effects included.^[^ ^125^, ^126^, ^127^ ^]^ The calculations suggest that this enhancement can be tentatively attributed to the presence of sulfur vacancies (*V*_S_) in 2L‐MoS_2_ (Figure 7d–f). *V*_S_ are found to be stable in their negative charge states (*V*_S_^–^) within the range of Fermi‐level values closer to the conduction‐band minimum (CBM). Under n‐type conditions, *V*_S_^−^ represents the lowest‐energy magnetic native point defect (1*µ*_B_ per *V*_S_^–^) (Figure 7e). The magnetic interaction between the two *V*_S_^−^ defects is weakly ferromagnetic. In the VSe_2_/MoS_2_ system, the presence of the VSe_2_ layer with a larger work function of ≈4.5 eV (compared to ≈4.1 eV for the MoS_2_ layer) results in an accumulation of electrons in the VSe_2_ layer, creating a depleted region in the MoS_2_ side of the hetero‐interface and subsequent formation of a Schottky barrier. The mechanism for ferromagnetism in the VSe_2_/MoS_2_ system remains under debate; however, the accumulation of electrons in the VSe_2_ layer could give rise to enhanced ferromagnetism in this layer and thus contribute to the change in net magnetization of the VSe_2_/MoS_2_ heterostructure when the material is not exposed to light. It should be noted that 1L‐VSe_2_ has a more dominant magnetic contribution to the net magnetization of the 1L‐VSe_2_/2L‐MoS_2_ film.

Upon light illumination, electron–hole pairs are generated and subsequently separated by the interfacial electric field at the Schottky barrier between the two materials. Consequently, an accumulation of excited electrons builds up in the conduction band of MoS_2_. This 2D electron gas can increase the negative charge of the sulfur vacancies in the MoS_2_ layer, which, in turn, increases the net magnetic moment (Figure 7f). For instance, the nonmagnetic *V*_S_^0^ becomes *V*_S_^−^, for a certain duration (during laser irradiation). Our calculations indicate that a portion of the extra electrons will localize on the vacancy. At higher laser powers, more photo‐generated electrons in the conduction band of MoS_2_ will completely fill the available confined‐states and eventually leak to the VSe_2_ layer, which could explain the saturation of magnetization enhancement. Due to the 2D nature of 2L‐MoS_2_, both photo‐generated electrons and sulfur vacancies are confined to the vicinity of the heterostructure interface. This facilitates the ferromagnetic interaction between nearby neighboring vacancies, which not only leads to a larger magnetic moment in the film but also enhances light‐controlled magnetism compared to magnetically doped TMD monolayers discussed previously. This suggests that besides the interfacial coupling of the heterostructure, the electron confinement and the concentration of sulfur vacancies also mediate the change in magnetization of the film under illumination (see, Figure 7a–c). This effect is significantly reduced when the thickness of the MoS_2_ layer is increased, as demonstrated by the change in magnetization of the BSC‐MoS_2_/VSe_2_ being four times smaller than that of 2L‐MoS_2_/VSe_2_ (Figure 7a). We attribute this to the lack of confinement near the MoS_2_/VSe_2_ interface. In the case of BSC‐MoS_2_/VSe_2_ (Figure 7c), photogenerated electrons are no longer confined to the interface but can move deeper into the MoS_2_ layer, significantly reducing the concentration of electrons near the interface. Nevertheless, further studies are required to fully understand the mechanism of light‐mediated ferromagnetism in the VSe_2_/MoS_2_ system. It should also be noted that sulfur vacancies in 2L‐MoS_2_ are likely not the sole source of magnetism; other point defects and extended defects in the samples may play a role. Our theoretical argument regarding light‐enhanced ferromagnetism is applicable to any defect whose electrical and magnetic properties resemble those of sulfur vacancies.

## 4 Opportunities and Challenges

Our findings have established that the light‐modulated magnetism effect is *universal* to 2D‐TMD DMSs, including V‐doped TMD monolayers (V‐WS_2_, V‐WSe_2_, and V‐MoS_2_). In addition to their electrically tunable magnetic functionalities, the optically tunable atomic‐level magnetism at room temperature makes 2D‐TMD DMSs even more attractive for applications in spintronics, spin‐caloritronics, and valleytronics. The coexistence of magnetic and semiconducting properties is necessary; therefore, higher doping concentrations are unlikely to exhibit this effect, as their semiconducting qualities are strongly suppressed. On the other hand, lower V‐doping concentrations are interesting to study, since excellent semiconducting properties are preserved while the magnetic moment is slightly smaller compared to optimally V‐doped TMD samples. Investigating the influence of different V‐doping concentrations on 2D‐TMDs’ light‐mediated magnetism effect is an intriguing direction for future research. Further studies are necessary to understand the relationship between doping concentration and light‐enhanced magnetism.

In addition, we have demonstrated the universality of the light‐mediated magnetism effect in 2D‐TMD ferromagnet/semiconductor heterostructures, including VSe_2_/WS_2_ and VSe_2_/MoS_2_ systems. Charge transfer, proximity, and confinement effects play a crucial role in enhancing light‐mediated magnetization in these 2D systems. However, other effects such as interdiffusion, intercalation, twisting, and moiré patterns, which could occur during heterostructure formation remain largely unexplored.^[^ ^31^, ^45^, ^51^, ^78^, ^103^, ^128^ ^]^ Recently, Wang et al. revealed the novel possibility of tuning spin‐spin interactions between moiré‐trapped holes using optical means, inducing a ferromagnetic order in WS_2_/WSe_2_ moiré superlattices.^[^ ^103^ ^]^ In the case of VSe_2_/MoS_2_ heterostructures, effects of the moiré pattern has been observed at low temperatures (below the charge density wave transition temperature, *T*_CDW_), leading to enhanced magnetization, strong interfacial magnetic coupling, and the exchange bias (EB) effect within this temperature range.^[^ ^13^, ^84^ ^]^ To fully understand the moiré effect, and to exploit the light tunability of the EB effect, it would be interesting to investigate the light‐mediated magnetism effect in VSe_2_/MoS_2_ and VSe_2_/WS_2_ heterostructures at *T* < *T_CDW_*. DFT calculations suggest that charge transfer from the ferromagnetic metal VSe_2_ (CrSe_2_) to the semiconductor MoS_2_ (WSe_2_) gives rise to the magnetic moment of the MoS_2_ (WSe_2_) layer. A fundamental question emerges: *Will a similar effect occur when the 2D semiconducting TMD (MoS_2_, WS_2_*, etc.*) layer interfaces with a non‐magnetic metal like graphene?* Exploring magnetism and light effects in semiconducting 2D‐TMDs (both pristine and magnetically doped TMDs) interfaced with graphene will not only address this important question but also provide new insights into charge transfer‐mediated magnetism in light‐illuminated van der Waals heterostructures composed of 2D‐TMD DMSs and other 2D materials. Depending on the work function difference between the two component materials, holes/electrons can be transferred into 2D‐TMD DMSs, resulting in enhanced or reduced magnetization. This represents a promising, innovative approach for designing novel 2D‐TMD heterostructures with enhanced magnetic and magneto‐optic properties through a combined chemical doping and interface engineering (via the light‐modulated directional charge transfer mechanism) strategy.

Proximity effects in 2D van der Waals materials benefit from ultraclean interfaces that are not accessible to 3D materials, and how they manifest is strongly dependent in the electronic properties at the interface. The magnetic proximity effect can manifest in a myriad of ways, most commonly by a magnetic material inducing magnetic ordering in a nearby non‐magnetic layer. Another important manifestation of the proximity effect is the induction and enhancement of spin–orbit coupling from the atomically heavy TMDs, onto a nearby layer with non‐existent or weak spin–orbit coupling.^[^ ^129^, ^130^ ^]^ During fabrication or stacking, the joint properties of the interface may change due to charge transfer,^[^ ^131^, ^132^ ^]^ wave function hybridization,^[^ ^133^ ^]^ changes in the band structure,^[^ ^134^, ^135^ ^]^ and lattice symmetry breaking,^[^ ^136^ ^]^ which has led to the discovery of new physics such as valleytronics, and has opened the door to a wide range of possible combinations waiting to be discovered. Several 2D TMD heterostructures have been studied to date and have been reviewed in previous publications, but continuing efforts in their discovery and characterization will lead to important technological advances in the semiconductor industry and beyond.^[^ ^7^, ^15^, ^137^, ^138^, ^139^, ^140^ ^]^

From an application perspective, the optically tunable magnetic properties of 2D‐TMD DMSs and heterostructures are desirable for opto‐spintronics, opto‐spin‐caloritronics, and valleytronic. Ghiasi et al. demonstrated charge‐to‐spin conversion across a monolayer WS_2_/graphene interface due to the Rashba‐Edelstein effect (REE).^[^ ^141^ ^]^ B. Zhao et al. similarly showed charge‐spin conversion in (20nm‐70 nm) WTe_2_/graphene heterostructure at room temperature, unconventionally mediated by a combined spin Hall effect and REE.^[^ ^142^ ^]^ Alternatively, using 2D‐TMD DMSs such as V‐WS_2_ and V‐WSe_2_ monolayers may not only boost spin‐charge conversion efficiency but also enable optical manipulation of the spin‐charge conversion process in a truly atomically thin spintronic device, that is compatible with existing Si‐based technology. To achieve this, it is imperative that research groups continue developing synthesis methods to increase yield and attain consistent doping uniformity across batches. A comprehensive review by Sierra et al. highlighted the novel application perspectives of opto‐spintronics.^[^ ^7^ ^]^

Thermally induced spin currents based on the spin Seebeck effect (SSE), a phenomenon discovered by Uchida et al.,^[^ ^143^, ^144^ ^]^ laid the foundation for a new generation of spin‐caloritronic devices. A pure spin current can be generated in a ferromagnetic (FM) material (like YIG: Y_3_Fe_5_O_12_) due to a built‐up electric potential across a temperature gradient upon the application of a magnetic field. This spin current can be converted into a technologically useful voltage via the inverse spin Hall effect of a heavy metal (HM) with strong spin‐orbit coupling (like Pt) in an FM/HM structure. Recently, Kalappattil et al. showed that inserting a thin (≈5 nm) organic *semiconducting* layer of C_60_ can significantly reduce the conductivity mismatch between YIG and Pt and the surface perpendicular magnetic anisotropy of YIG, resulting in a giant enhancement (600%) in the longitudinal SSE.^[^ ^95^ ^]^ Following this approach, Lee et al. inserted a *semiconducting* WSe_2_ monolayer between Pt and YIG and observed the giant SSE in Pt/WSe_2_/YIG.^[^ ^145^ ^]^ DFT calculations indicate that inserting a 2D‐TMD DMS (e.g., V‐WSe_2_) in an FM/HM bilayer system not only reduces the conductivity mismatch but also enhances the spin mixing conductance and hence the spin‐to‐charge conversion efficiency via the SSE.^[^ ^145^ ^]^ By taking advantage of the light‐tunable magnetization of 2D‐TMD DMSs,^[^ ^56^, ^94^ ^]^ Phan et al. recently proposed a new route for the optical control of thermally induced spin currents through 2D‐TMD DMS interfaces in FM/HM systems, establishing the new subfield named “Opto‐spin‐caloritronics”,^[^ ^83^ ^]^ which can harness “*light as the new heat*”. Further studies are needed to fully exploit this potential.

**Scheme 1:**
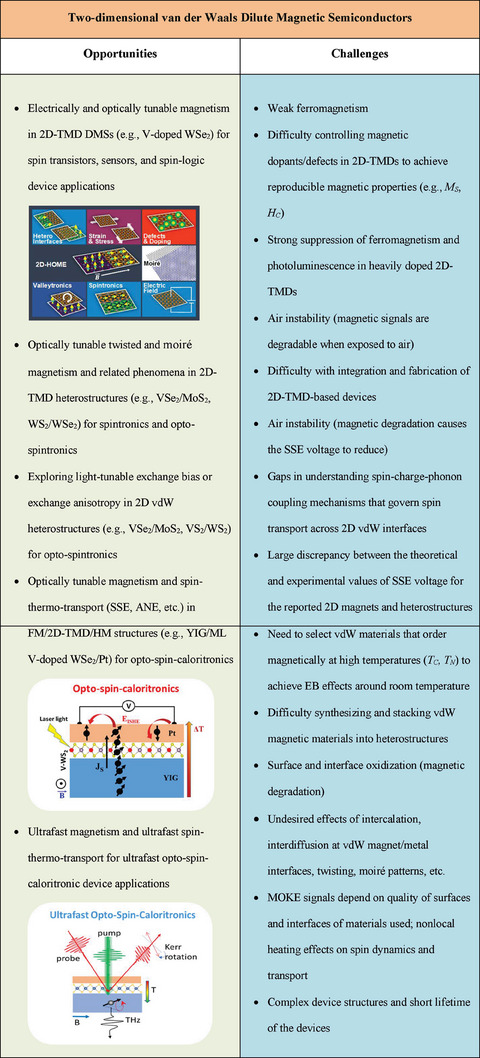
Opportunities and challenges in the research of 2D‐TMD magnets.

Semiconducting 2D‐TMDs (e.g., WSe_2_, WS_2_, MoS_2_) are excellent candidates for use in valleytronic devices.^[^ ^48^, ^49^ ^]^ Controlling and manipulating the valley polarization states in these 2D‐TMDs using external stimuli (optic, electric, and magnetic fields) is essential.^[^ ^77^, ^146^, ^147^, ^148^, ^149^ ^]^ Doping magnetic atoms (Fe, V) into a MoS_2_ monolayer to form Fe‐MoS_2_ or V‐MoS_2_ DMSs has been reported to enhance valley splitting in MoS_2_ monolayers.^[^ ^66^, ^67^ ^]^ Since 2D‐TMD DMSs exhibit strong magnetic responses to both electric fields and lasers,^[^ ^56^, ^57^ ^]^ their valleytronic properties can be manipulated by these external stimuli. Seyler et al. experimentally exploited light to control CrI_3_ magnetization in a CrI_3_/WSe_2_ heterostructure, demonstrating the optical modulation of valley polarization and valley Zeeman splitting within the WSe_2_ monolayer.^[^ ^41^ ^]^ However, the CrI_3_ monolayer exhibits ferromagnetic ordering below 50 K, rendering the CrI_3_/WSe_2_ heterostructure impractical for use in valleytronic devices that operate at ambient temperatures. In this context, the optical modulation of magnetic and valleytronic properties of VSe_2_(MnSe_2_)/MoS_2_ and VSe_2_(MnSe_2_)/WS_2_ heterostructures appears more compelling, as the VSe_2_ or MnSe_2_ layer exhibits ferromagnetic order above room temperature.^[^ ^13^, ^14^ ^]^ Based on DFT calculations, He et al. showed that an ultrafast laser pulse can induce a ferromagnetic state in a nonmagnetic MoSe_2_ monolayer when interfaced with the MnSe_2_ monolayer, which orders ferromagnetically above room temperature.^[^ ^150^ ^]^ Such ultrafast optical control of 2D magnetism is highly compelling for applications in ultrafast spintronics and magnetic storage information technology.^[^ ^151^ ^]^ It is worth noting that the magnetic properties of 2D‐TMDs have contributions from defect‐ and dopant‐induced magnetic moments and their couplings.^[^ ^51^, ^60^ ^]^ Therefore, it would be of significant interest to investigate the effects of transition metal or chalcogen vacancies and magnetic dopant concentrations on the magnetic, magneto‐optic, and valleytronic properties of free‐standing TMD monolayers, as well as those placed on magnetic substrates in heterostructures. **Scheme** **1** highlights opportunities and challenges in exploiting 2D‐TMD DMSs and heterostructures for use in modern nanodevices.

## 5 Concluding Remarks and Outlook

We have established the universality of the light‐modulated magnetization effect in 2D‐TMD DMSs, including V‐doped TMD monolayers (V‐WS_2_, V‐WSe_2_, V‐MoS_2_). This effect is attributed to the presence of excess holes in the conduction and valence bands, as well as carriers trapped in the magnetic doping states, which mediate the magnetization of the TMD layer. Additionally, we have demonstrated the universality of the light‐mediated magnetism effect in 2D‐TMD ferromagnet/semiconductor heterostructures such as VSe_2_/WS_2_ and VSe_2_/MoS_2_. This effect is attributed to photon absorption at the TMD layer (e.g., WS_2_, WSe_2_, MoS_2_), generating electron–hole pairs that mediate the magnetization of the heterostructure. These findings pave a new pathway for the design of novel 2D‐TMD van der Waals heterostructures that exhibit unique magneto‐optical coupling functionalities that enable the next generation of high‐performance optoelectronics, ultrafast opto‐spintronics, opto‐spin‐caloritronics, valleytronics, and quantum technologies.

We have demonstrated the importance of proximity, charge‐transfer, and confinement effects in enhancing light‐mediated magnetization in 2D‐TMD heterostructures, but other effects such as interdiffusion, intercalation, twisting, and moiré patterns may also be significant.^[^ ^31^, ^45^, ^51^, ^78^, ^103^, ^128^ ^]^ Further studies are thus needed to fully understand these effects. It appears that 2D‐TMD magnetism arises from multiple contributions of vacancy‐ and dopant‐induced magnetic moments, as well as their magnetic couplings, whose strengths vary depending on their complex vacancy‐dopant configurations.^[^ ^51^, ^60^ ^]^ Understanding how light mediates magnetization in 2D‐TMDs with controlled dopant/vacancy concentrations is critical. The “twisting” effect has been reported to significantly alter the magnetic and valleytronic properties of 2D‐TMDs.^[^ ^129^, ^152^, ^153^, ^154^, ^155^, ^156^ ^]^ Twisting graphene from a 2D‐TMD in a 2D‐TMD/graphene heterostructure can enhance the valley Zeeman and Rashba effects,^[^ ^129^, ^152^ ^]^ as well as the charge‐to‐spin conversion efficiency.^[^ ^153^, ^154^ ^]^ By tailoring the atomic interface between twisted bilayer graphene and WSe_2_, Lin et al. showed strong electron correlation within the moiré flat band, which stabilizes insulating states at both quarter and half filling, and the spin‐orbit coupling drives the Mott‐like insulator into ferromagnetism.^[^ ^155^ ^]^ In addition to the magnetic proximity and charge transfer effects, twisting adds an interesting experimental knob to tune the magnetic and magneto‐optic functionalities of 2D‐TMDs for spintronics and valleytronics applications. Continuing efforts in discovering new combinations of TMD heterostructures, as well as improved growth and device fabrication processes are imperative to push the field forward.

From an application standpoint, the room‐temperature electrically and optically tunable magnetic properties make 2D‐TMD DMSs excellent candidates for use in spin transistors, logic, and magnetic memory devices.^[^ ^51^, ^54^, ^85^ ^]^ In opto‐spintronics, ultrafast optical control of 2D magnetization may yield the fastest information recording and processing with minimal dissipative power.^[^ ^7^, ^155^ ^]^ Experimental studies are needed to verify these theoretical predictions.^[^ ^73^, ^122^ ^]^ Additionally, 2D‐TMD DMSs can serve as novel 2D spin filters to boost the spin‐to‐charge conversion efficiency via the SSE in FM/2D‐DMS/HM systems.^[^ ^83^, ^118^ ^]^ A comprehensive understanding of the spin‐charge‐phonon coupling mechanisms in such 2D spin filters is currently lacking but crucial for unlocking the potential of “Opto‐Spin‐Caloritronics,” which warrants further study.

Compared to their 2D‐TMD counterparts, 2D‐TMD DMSs appear more promising for use in valleytronic devices.^[^ ^66^, ^67^ ^]^ To further enhance the valley splitting in these 2D DMSs, it is possible to interface them with other 2D materials such as graphene. Combining chemical doping and interface engineering (charge transfer and/or strain) approaches can create 2D‐TMD DMS/graphene or 2D‐TMD DMS/2D‐TMD heterostructures with enhanced magnetic, magneto‐optic, and valleytronic properties that can be tuned by external stimuli (electric gating, light, and strain). All these exciting possibilities will facilitate further research.

To provide insightful guidance on the development of 2D‐TMD‐based devices, we present in **Table** **1** a list of promising 2D‐TMD magnets and heterostructures. While most of the 2D‐TMD DMSs are synthesized using chemical vapor deposition (CVD), some of their heterostructures are grown by molecular beam epitaxy (MBE) or a combination of both techniques.^[^ ^76^ ^]^ CVD typically produces 2D films with uniformity, low porosity, high purity, and stability, but generates toxic gases during the reaction. MBE enables in‐situ preparation of atomically clean substrates with specific surface reconstructions, facilitating the growth of highly epitaxial 2D films, but it can result in more defects during film growth.^[^ ^74^, ^75^, ^76^ ^]^ It is essential to advance these techniques for growing defect‐free or defect‐controllable 2D‐TMD DMSs and heterostructures.

**Table 1:** 2D‐TMD magnets and heterostructures with optically tunable magnetic functionalities for spintronics, spin‐caloritronics, and valleytronics. Legend for remarks: ♦ Unexplored optical control of magnetism and valleytronic states; ↔ Charge transfer; ⊗ Less air stability; ♣ Less sensitive to air; ♥ Air stability.

Materials	Ordering temperature *T*_C_ (K)	Remarks	Ref.
Metals			
VSe_2_ (1L, 2L)	≈270–350 K	Strong magnetism; Sensitive to defects;	[13]
MnSe_2_ (1L, 2L)	≈266–350 K	Intrinsic magnetism; Less sensitive to defects; ⊗	[14]
CrSe_2_ (1L)	50–300 K	Intrinsic magnetism; Less sensitive to defects; ♥	[50, 157]
FeSe_2_ (1L)	≈300 K	Intrinsic magnetism; ♥	[156]
Semiconductors			
V‐WS_2_ (1L) optimal ≈2 at.%	≈300–400 K	♦; ♣	[52]
V‐WSe_2_ (1L) optimal ≈4 at.%	≈300–400 K	♦; ♣	[53]
V‐MoSe_2_ (1L) optimal ≈2 at.%	≈300–400 K	♦; ♣	[63]
V‐MoS_2_ (1L)	≈300–400 K	♦; ♣	[61, 67]
V‐MoTe_2_ (1L)	≈300–400 K	♦; ♣	[70]
Fe‐MoS_2_ (1L)	≈300–400 K	♦; ♣	[55, 66]
Co‐MoS_2_ (1L)	≈300 K	♦; ♣	[58]
(Co,Cr)‐MoS_2_ (1L)	≈300 K	♦; ♣	[71]
Co‐SnS_2_ (SC)	≈120 K	♦; ♣	[159]
Fe‐SnS_2_ (1L)	≈30 K	♦;♥	[160]
Mn‐SnS_2_ (SC)	≈150 K	♦;♥	[161]
Heterostructures			
CrSe_2_/WSe_2_ (1L/1L)	≈50–120 K	↔;♦; ♣	[50]
VSe_2_/MoTe_2_ (1L/1L)	≈300–350 K	↔;♦; ♣	[120]
VSe_2_/MoS_2_ (1L/2L)	≈300–350 K	↔;♦; ♣; Observed EB effect;	[13, 84]
MnSe_2_/MoSe_2_ (1L/1L)	≈300–350 K	↔;♦; ♣	[121]
VS_2_/MoS_2_ (1L/1L)	≈300–350 K	↔;♦; ♣	[158]

As noted earlier, the high concentrations of magnetic dopants or the presence of abundant defects (transition metal/chalcogen vacancies) in 2D‐TMD semiconductors lead to the strong suppression of photoluminescence.^[^ ^51^, ^52^, ^53^ ^]^ However, co‐doping with different metals such as, Co and Cr, in a MoS_2_ monolayer has been reported to enhance both photoluminescence intensity and saturation magnetization.^[^ ^71^ ^]^ Combining chemical co‐doping and interface engineering represents a promising strategy for the design of 2D‐TMD DMSs with enhanced magnetic and magneto‐optic properties for spintronics, opto‐spintronics, opto‐spin‐caloritronics, valleytronics, and quantum communications.

## Conflict of Interest

The authors declare no conflicts of interest.

## Author Contributions

V.O.J., M.T., and M.H.P. initiated the research. V.O.J. performed light‐mediated magnetism experiments and analyzed data. Y.T.H.P., V.K., and T.E. performed magnetic measurements and analysis. D.Z. M.Z.L., F.A.N., M.T., and H.R.G. were involved in the synthesis of the TMD samples and participated in STEM experiments and analysis. K.H. and D.L.D. performed the DFT calculations. V.O.J., K.H., and M.H.P. prepared the first draft of the manuscript and all the co‐authors contributed to the final version of the manuscript. M.H.P. led the project.

## References

[1] P. Chuang , S.‐C. Ho , L. W. Smith , F. Sfigakis , M. Pepper , C.‐H. Chen , J.‐C. Fan , J. P. Griffiths , I. Farrer , H. E. Beere , G. A. C. Jones , D. A. Ritchie , T.‐M. Chen , Nat. Nanotechnol. 2015, 10, 35. 25531088 10.1038/nnano.2014.296

[2] F. Matsukura , Y. Tokura , H. Ohno , Nat. Nanotechnol. 2015, 10, 209. 25740132 10.1038/nnano.2015.22

[3] W. Yan , O. Txoperena , R. Llopis , H. Dery , L. E. Hueso , F. Casanova , Nat. Commun. 2016, 7, 13372. 27834365 10.1038/ncomms13372 PMC5114593

[4] B. Dieny , I. L. Prejbeanu , K. Garello , P. Gambardella , P. Freitas , R. Lehndorff , W. Raberg , U. Ebels , S. O. Demokritov , J. Akerman , A. Deac , P. Pirro , C. Adelmann , A. Anane , A. A. Chumak , A. Hirohata , S. Mangin , S. O. Valenzuela , M. C. Onbasli , M. d'Aquino , G. Prenat , G. Finocchio , L. Lopez‐Diaz , R. Chantrell , O. Chubykalo‐Fresenko , P. Bortolotti , Nat. Electron. 2020, 3, 446.

[5] G. F. A. Malik , M. A. Kharadi , F. A. Khanday , N. Parveen , Microelectron. J. 2020, 106, 104924.

[6] J. Ingla‐Aynés , F. Herling , J. Fabian , L. E. Hueso , F. Casanova , Phys. Rev. Lett. 2021, 127, 047202. 34355972 10.1103/PhysRevLett.127.047202

[7] J. F. Sierra , J. Fabian , R. K. Kawakami , S. Roche , S. O. Valenzuela , Nat. Nanotechnol. 2021, 16, 856. 34282312 10.1038/s41565-021-00936-x

[8] A. Avsar , H. Ochoa , F. Guinea , B. Özyilmaz , B. ? J. Van Wees , I. J. Vera‐Marun , Rev. Mod. Phys. 2020, 92, 021003.

[9] E. C. Ahn , npj 2D Mater. Appl. 2020, 4, 17.

[10] B. Huang , G. Clark , E. Navarro‐Moratalla , D. R. Klein , R. Cheng , K. L. Seyler , D. Zhong , E. Schmidgall , M. A. Mcguire , D. H. Cobden , W. Yao , D. Xiao , P. Jarillo‐Herrero , X. Xu , Nature 2017, 546, 270. 28593970 10.1038/nature22391

[11] C. Gong , L. Li , Z. Li , H. Ji , A. Stern , Y. Xia , T. Cao , W. Bao , C. Wang , Y. Wang , Z. Q. Qiu , R. J. Cava , S. G. Louie , J. Xia , X. Zhang , Nature 2017, 546, 265. 28445468 10.1038/nature22060

[12] Z. Fei , B. Huang , P. Malinowski , W. Wang , T. Song , J. Sanchez , W. Yao , D. Xiao , X. Zhu , A. F. May , W. Wu , D. H. Cobden , J.‐H. Chu , X. Xu , Nat. Mater. 2018, 17, 778. 30104669 10.1038/s41563-018-0149-7

[13] M. Bonilla , S. Kolekar , Y. Ma , H. C. Diaz , V. Kalappattil , R. Das , T. Eggers , H. R. Gutierrez , M.‐H. Phan , M. Batzill , Nat. Nanotechnol. 2018, 13, 289. 29459653 10.1038/s41565-018-0063-9

[14] D. J. O'hara , T. Zhu , A. H. Trout , A. S. Ahmed , Y. K. Luo , C. H. Lee , M. R. Brenner , S. Rajan , J. A. Gupta , D. W. Mccomb , R. K. Kawakami , Nano Lett. 2018, 18, 3125. 29608316 10.1021/acs.nanolett.8b00683

[15] W. Li , Y. Zeng , Z. Zhao , B. Zhang , J. Xu , X. Huang , Y. Hou , ACS Appl. Mater. Interfaces 2021, 13, 50591. 34674524 10.1021/acsami.1c11132

[16] G. Hu , B. Xiang , Nanoscale Res. Lett. 2020, 15, 226. 33296058 10.1186/s11671-020-03458-y PMC7726086

[17] T. Vincent , J. Liang , S. Singh , E. G. Castanon , X. Zhang , A. Mccreary , D. Jariwala , O. Kazakova , Z. Y. Al Balushi , Appl. Phys. Rev. 2021, 8, 041320.

[18] Y. Li , B. Yang , S. Xu , B. Huang , W. Duan , ACS Appl. Electron. Mater. 2022, 4, 3278.

[19] H. Kurebayashi , J. H. Garcia , S. Khan , J. Sinova , S. Roche , Nat. Rev. Phys. 2022, 4, 150.

[20] M. Gibertini , M. Koperski , A. F. Morpurgo , K. S. Novoselov , Nat. Nanotechnol. 2019, 14, 408. 31065072 10.1038/s41565-019-0438-6

[21] Y. Khan , S. M. Obaidulla , M. R. Habib , A. Gayen , T. Liang , X. Wang , M. Xu , Nano Today 2020, 34, 100902.

[22] X. Jiang , Q. Liu , J. Xing , N. Liu , Y. Guo , Z. Liu , J. Zhao , Appl. Phys. Rev. 2021, 8, 031305.

[23] S. Zhang , R. Xu , N. Luo , X. Zou , Nanoscale 2021, 13, 1398. 33416064 10.1039/d0nr06813f

[24] W. Tang , H. Liu , Z. Li , A. Pan , Y.‐J. Zeng , Adv. Sci. 2021, 8, 2100847. 10.1002/advs.202100847 PMC8456225 34323390

[25] Y. Liu , Q. Shao , ACS Nano 2020, 14, 9389. 32692151 10.1021/acsnano.0c04403

[26] J.‐F. Dayen , S. J. Ray , O. Karis , I. J. Vera‐Marun , M. V. Kamalakar , Appl. Phys. Rev. 2020, 7, 011303.

[27] M.‐H. Phan , V. Kalappattil , V. O. Jimenez , Y. Thi Hai Pham , N. W. Y. A. Y. Mudiyanselage , D. Detellem , C.‐M. Hung , A. Chanda , T. Eggers , J. Alloys Compd. 2023, 937, 168375.

[28] C. Lei , B. L. Chittari , K. Nomura , N. Banerjee , J. Jung , A. H. Macdonald , Nano Lett. 2021, 21, 1948. 33600723 10.1021/acs.nanolett.0c04242

[29] S. Jiang , J. Shan , K. F. Mak , Nat. Mater. 2018, 17, 406. 29531370 10.1038/s41563-018-0040-6

[30] B. Huang , G. Clark , D. R. Klein , D. Macneill , E. Navarro‐Moratalla , K. L. Seyler , N. Wilson , M. A. Mcguire , D. H. Cobden , D. Xiao , W. Yao , P. Jarillo‐Herrero , X. Xu , Nat. Nanotechnol. 2018, 13, 544. 29686292 10.1038/s41565-018-0121-3

[31] Y. Xu , A. Ray , Y.‐T. Shao , S. Jiang , K. Lee , D. Weber , J. E. Goldberger , K. Watanabe , T. Taniguchi , D. A. Muller , K. F. Mak , J. Shan , Nat. Nanotechnol. 2022, 17, 143. 34845332 10.1038/s41565-021-01014-y

[32] I. A. Verzhbitskiy , H. Kurebayashi , H. Cheng , J. Zhou , S. Khan , Y. P. Feng , G. Eda , Nat. Electron. 2020, 3, 460.

[33] W. Zhuo , B. Lei , S. Wu , F. Yu , C. Zhu , J. Cui , Z. Sun , D. Ma , M. Shi , H. Wang , W. Wang , T. Wu , J. Ying , S. Wu , Z. Wang , X. Chen , M. , Adv. Mater. 2021, 33, 2008586. 10.1002/adma.202008586 34173269

[34] Z. Wang , T. Zhang , M. Ding , B. Dong , Y. Li , M. Chen , X. Li , J. Huang , H. Wang , X. Zhao , Y. Li , D. Li , C. Jia , L. Sun , H. Guo , Y. Ye , D. Sun , Y. Chen , T. Yang , J. Zhang , S. Ono , Z. Han , Z. Zhang , Nat. Nanotechnol. 2018, 13, 554. 29967458 10.1038/s41565-018-0186-z

[35] V. Ostwal , T. Shen , J. Appenzeller , Adv. Mater. 2020, 32, 1906021. 10.1002/adma.201906021 31930776

[36] A. Ilyas , S. Xiang , M. Chen , M. Y. Khan , H. Bai , P. He , Y. Lu , R. Deng , Nanoscale 2021, 13, 1069. 33393568 10.1039/d0nr06054b

[37] V. Gupta , T. M. Cham , G. M. Stiehl , A. Bose , J. A. Mittelstaedt , K. Kang , S. Jiang , K. F. Mak , J. Shan , R. A. Buhrman , D. C. Ralph , Nano Lett. 2020, 20, 7482. 32975955 10.1021/acs.nanolett.0c02965

[38] Y. Wu , B. Francisco , Z. Chen , W. Wang , Y. Zhang , C. Wan , X. Han , H. Chi , Y. Hou , A. Lodesani , G. Yin , K. Liu , Y.‐T. Cui , K. L. Wang , J. S. Moodera , Adv. Mater. 2022, 34, 2110583. 10.1002/adma.202110583 35218078

[39] P. Zhang , T.‐F. Chung , Q. Li , S. Wang , Q. Wang , W. L. B. Huey , S. Yang , J. E. Goldberger , J. Yao , X. Zhang , Nat. Mater. 2022, 21, 1373. 36109674 10.1038/s41563-022-01354-7

[40] T. Zhang , Y. Chen , Y. Li , Z. Guo , Z. Wang , Z. Han , W. He , J. Zhang , Appl. Phys. Lett. 2020, 116, 223103.

[41] K. L. Seyler , D. Zhong , B. Huang , X. Linpeng , N. P. Wilson , T. Taniguchi , K. Watanabe , W. Yao , D. Xiao , M. A. Mcguire , K.‐M. C. Fu , X. Xu , Nano. Lett. 2018, 18, 3823. 29756784 10.1021/acs.nanolett.8b01105

[42] R. Zhu , W. Zhang , W. Shen , P. K. J. Wong , Q. Wang , Q. Liang , Z. Tian , Y. Zhai , C.‐W. Qiu , A. T. S. Wee , Nano. Lett. 2020, 20, 5030. 32463247 10.1021/acs.nanolett.0c01149

[43] M. DaBrowski , S. Guo , M. Strungaru , P. S. Keatley , F. Withers , E. J. G. Santos , R. J. Hicken , Nat. Commun. 2022, 13, 5976. 36216796 10.1038/s41467-022-33343-4 PMC9551086

[44] Z. Zhang , X. Ni , H. Huang , L. Hu , F. Liu , Phys. Rev. B 2019, 99, 115441.

[45] M. Ge , H. Wang , J. Wu , C. Si , J. Zhang , S. Zhang , npj Comput. Mater. 2022, 8, 32.

[46] T. Zhang , S. Zhao , A. Wang , Z. Xiong , Y. Liu , M. Xi , S. Li , H. Lei , Z. V. Han , F. Wang , Adv. Funct. Mater. 2022, 32, 2204779.

[47] B. Marfoua , J. Hong , Nanotechnology 2020, 31, 425702. 32599576 10.1088/1361-6528/aba0f4

[48] S. A. Vitale , D. Nezich , J. O. Varghese , P. Kim , N. Gedik , P. Jarillo‐Herrero , D. Xiao , M. Rothschild , Small 2018, 14, 1801483. 10.1002/smll.201801483 30102452

[49] K. F. Mak , D. Xiao , J. Shan , Nat. Photonics 2018, 12, 451.

[50] B. Li , Z. Wan , C. Wang , P. Chen , B. Huang , X. Cheng , Q. Qian , J. Li , Z. Zhang , G. Sun , B. Zhao , H. Ma , R. Wu , Z. Wei , Y. Liu , L. Liao , Y. Ye , Y. Huang , X. Xu , X. Duan , W. Ji , X. Duan , Nat. Mater. 2021, 20, 818. 33649563 10.1038/s41563-021-00927-2

[51] Y. L. Huang , W. Chen , A. T. S. Wee , SmartMat 2021, 2, 139.

[52] F. Zhang , B. Zheng , A. Sebastian , D. H. Olson , M. Liu , K. Fujisawa , Y. T. H. Pham , V. O. Jimenez , V. Kalappattil , L. Miao , T. Zhang , R. Pendurthi , Y. Lei , A. L. Elías , Y. Wang , N. Alem , P. E. Hopkins , S. Das , V. H. Crespi , M.‐H. Phan , M. Terrones , Adv. Sci. 2020, 7, 2001174. 10.1002/advs.202001174 PMC7740087 33344114

[53] Y. T. H. Pham , M. Liu , V. O. Jimenez , Z. Yu , V. Kalappattil , F. Zhang , K. Wang , T. Williams , M. Terrones , M.‐H. Phan , Adv. Mater. 2020, 32, 2003607. 10.1002/adma.202003607 33015889

[54] S. J. Yun , D. L. Duong , D. M. Ha , K. Singh , T. L. Phan , W. Choi , Y.‐M. Kim , Y. H. Lee , Adv. Sci. 2020, 7, 1903076. 10.1002/advs.201903076 PMC7201245 32382479

[55] S. Fu , K. Kang , K. Shayan , A. Yoshimura , S. Dadras , X. Wang , L. Zhang , S. Chen , N. Liu , A. Jindal , X. Li , A. N. Pasupathy , A. N. Vamivakas , V. Meunier , S. Strauf , E.‐H. Yang , Nat. Commun. 2020, 11, 2034. 32341412 10.1038/s41467-020-15877-7 PMC7184740

[56] V. Ortiz Jimenez , Y. T. H. Pham , M. Liu , F. Zhang , Z. Yu , V. Kalappattil , B. Muchharla , T. Eggers , D. L. Duong , M. Terrones , M.‐H. Phan , Adv. Electron. Mater. 2021, 7, 2100030.

[57] D. L. Duong , S.‐G. Kim , Y. H. Lee , AIP Adv. 2020, 10, 065220.

[58] M. Huang , J. Xiang , C. Feng , H. Huang , P. Liu , Y. Wu , A. T. N'Diaye , G. Chen , J. Liang , H. Yang , J. Liang , X. Cui , J. Zhang , Y. Lu , K. Liu , B. Xiang , ACS Appl. Electron. Mater. 2020, 2, 1497.

[59] L.‐A. T. Nguyen , K. P. Dhakal , Y. Lee , W. Choi , T. D. Nguyen , C. Hong , D. H. Luong , Y.‐M. Kim , J. Kim , M. Lee , T. Choi , A. J. Heinrich , J.‐H. Kim , D. Lee , D. L. Duong , Y. H. Lee , ACS Nano 2021, 15, 20267. 34807575 10.1021/acsnano.1c08375

[60] S. J. Yun , B. W. Cho , T. Dinesh , D. H. Yang , Y. I. Kim , J. W. Jin , S.‐H. Yang , T. D. Nguyen , Y.‐M. Kim , K. K. Kim , D. L. Duong , S.‐G. Kim , Y. H. Lee , Adv. Mater. 2022, 34, 2106551. 10.1002/adma.202106551 34962658

[61] J. Seo , E. Son , J. Kim , S.‐W. Kim , J. M. Baik , H. Park , Nano Res. 2023, 16, 3415.

[62] S. Stolz , A. Kozhakhmetov , C. Dong , O. Gröning , J. A. Robinson , B. Schuler , npj 2D Mater. Appl. 2022, 6, 66.

[63] J. Deng , Z. Zhou , J. Chen , Z. Cheng , J. Liu , Z. Wang , ChemPhysChem 2022, 23, 202200162. 10.1002/cphc.202200162 35593048

[64] J. Zhang , Y. Zhu , M. Tebyetekerwa , D. Li , D. Liu , W. Lei , L. Wang , Y. Zhang , Y. Lu , ACS Appl. Nano Mater. 2021, 4, 769.

[65] D. Shen , B. Zhao , Z. Zhang , H. Zhang , X. Yang , Z. Huang , B. Li , R. Song , Y. Jin , R. Wu , B. Li , J. Li , X. Duan , ACS Nano 2022, 16, 10623. 35735791 10.1021/acsnano.2c02214

[66] Q. Li , X. Zhao , L. Deng , Z. Shi , S. Liu , Q. Wei , L. Zhang , Y. Cheng , L. Zhang , H. Lu , W. Gao , W. Huang , C.‐W. Qiu , G. Xiang , S. J. Pennycook , Q. Xiong , K. P. Loh , B. Peng , ACS Nano 2020, 14, 4636. 32167276 10.1021/acsnano.0c00291

[67] K. R. Sahoo , J. J. Panda , S. Bawari , R. Sharma , D. Maity , A. Lal , R. Arenal , G. Rajalaksmi , T. N. Narayanan , Phys. Rev. Mater. 2022, 6, 085202.

[68] H. Shu , P. Luo , P. Liang , D. Cao , X. Chen , ACS Appl. Mater. Interfaces 2015, 7, 7534. 25805357 10.1021/am508843z

[69] B. Li , T. Xing , M. Zhong , L. Huang , N. Lei , J. Zhang , J. Li , Z. Wei , Nat. Commun. 2017, 8, 1958. 29208966 10.1038/s41467-017-02077-z PMC5717146

[70] P. M. Coelho , H.‐P. Komsa , K. Lasek , V. Kalappattil , J. Karthikeyan , M.‐H. Phan , A. V. Krasheninnikov , M. Batzill , Adv. Electron. Mater. 2019, 5, 1900044.

[71] H. Duan , P. Guo , C. Wang , H. Tan , W. Hu , W. Yan , C. Ma , L. Cai , L. Song , W. Zhang , Z. Sun , L. Wang , W. Zhao , Y. Yin , X. Li , S. Wei , Nat. Commun. 2019, 10, 1584. 30952850 10.1038/s41467-019-09531-0 PMC6451016

[72] L. Cai , V. Tung , A. Wee , J. Alloys Compd. 2022, 913, 165289.

[73] Y. Xiong , D. Xu , Y. Feng , G. Zhang , P. Lin , X. Chen , Adv. Mater. 2023, 2206939. 10.1002/adma.202206939 36245325

[74] L. Loh , Z. Zhang , M. Bosman , G. Eda , Nano Res. 2021, 14, 1668.

[75] Z. Zhao , W. Li , Y. Zheng , X. Huang , C. Yun , B. Zhang , Y. Hou , Small Struct. 2021, 2, 2100077.

[76] Y.‐C. Lin , R. Torsi , D. B. Geohegan , J. A. Robinson , K. Xiao , Adv. Sci. 2021, 8, 2004249. 10.1002/advs.202004249 PMC8097379 33977064

[77] J.‐X. Li , W.‐Q. Li , S.‐H. Hung , P.‐L. Chen , Y.‐C. Yang , T.‐Y. Chang , P.‐W. Chiu , H.‐T. Jeng , C.‐H. Liu , Nat. Nanotechnol. 2022, 17, 721. 35501377 10.1038/s41565-022-01115-2

[78] K. Zollner , P. E. Faria Junior , J. Fabian , Phys. Rev. B 2019, 100, 085128.

[79] C. Zhao , T. Norden , P. Zhang , P. Zhao , Y. Cheng , F. Sun , J. P. Parry , P. Taheri , J. Wang , Y. Yang , T. Scrace , K. Kang , S. Yang , G.‐X. Miao , R. Sabirianov , G. Kioseoglou , W. Huang , A. Petrou , H. Zeng , Nat. Nano. 2017, 12, 757. 10.1038/nnano.2017.68 28459469

[80] I. Khan , J. Ahmad , M. E. Mazhar , J. Hong , Nanotechnology 2021, 32, 375708. 10.1088/1361-6528/ac05e9 34044383

[81] C.‐M. Hung , D. T.‐X. Dang , A. Chanda , D. Detellem , N. Alzahrani , N. Kapuruge , Y. T. H. Pham , M. Liu , D. Zhou , H. R. Gutierrez , D. A. Arena , M. Terrones , S. Witanachchi , L. M. Woods , H. Srikanth , M.‐H. Phan , Nanomaterials 2023, 13, 771. 36839139 10.3390/nano13040771 PMC9967397

[82] J. Chu , Y. Wang , X. Wang , K. Hu , G. Rao , C. Gong , C. Wu , H. Hong , X. Wang , K. Liu , C. Gao , J. Xiong , Adv. Mater. 2021, 33, 2004469. 10.1002/adma.202004469 33325574

[83] M.‐H. Phan , M. T. Trinh , T. Eggers , V. Kalappattil , K.‐I. Uchida , L. M. Woods , M. Terrones , Appl. Phys. Lett. 2021, 119, 250501.

[84] X. Li , J. Yang , H. Sun , L. Huang , H. Li , J. Shi , Adv. Mater. 2023, 2305115. 10.1002/adma.202305115 37406665

[85] M. H. Phan , arXiv 2023.

[86] B. Xia , Y. Yang , J. Ma , K. Tao , D. Gao , Appl. Phys. Express 2017, 10, 093002.

[87] R. Zhang , Y. Du , G. Han , X. Gao , J. Mater. Sci. 2019, 54, 552.

[88] T. Sun , Z. Tang , W. Zang , Z. Li , J. Li , Z. Li , L. Cao , J. S. Dominic Rodriguez , C. O. M. Mariano , H. Xu , P. Lyu , X. Hai , H. Lin , X. Sheng , J. Shi , Y. Zheng , Y.‐R. Lu , Q. He , J. Chen , K. S. Novoselov , C.‐H. Chuang , S. Xi , X. Luo , J. Lu , Nat. Nanotechn. 2023, 18, 763. 10.1038/s41565-023-01407-1 37231143

[89] H. Liu , D. Fu , X. Li , J. Han , X. Chen , X. Wu , B. Sun , W. Tang , C. Ke , Y. Wu , Z. Wu , J. Kang , ACS Nano 2021, 15, 8244. 33982558 10.1021/acsnano.0c08305

[90] W. Hu , H. Tan , H. Duan , G. Li , N. Li , Q. Ji , Y. Lu , Y. Wang , Z. Sun , F. Hu , C. Wang , W. Yan , ACS Appl. Mater. Interfaces 2019, 11, 31155. 31385491 10.1021/acsami.9b09165

[91] W. S. Yun , J. D. Lee , J. Phys. Chem. C 2015, 119, 2822.

[92] F. Yang , P. Hu , F. F. Yang , B. Chen , F. Yin , R. Sun , K. Hao , F. Zhu , K. Wang , Z. Yin , Adv. Sci. 2023, 10, 2300952. 10.1002/advs.202300952 PMC10375142 37178366

[93] L.‐A. T. Nguyen , J. Jiang , T. D. Nguyen , P. Kim , M.‐K. Joo , D. L. Duong , Y. H. Lee , Nat. Electron. 2023, 6, 582.

[94] V. Ortiz Jimenez , Doctoral Thesis, University of South Florida 2022.

[95] V. Kalappattil , R. Geng , R. Das , M. Pham , H. Luong , T. Nguyen , A. Popescu , L. M. Woods , M. Kläui , H. Srikanth , M. H. Phan , Mater. Horizons. 2020, 7, 1413.

[96] B. Liu , S. Liu , L. Yang , Z. Chen , E. Zhang , Z. Li , J. Wu , X. Ruan , F. Xiu , W. Liu , L. He , R. Zhang , Y. Xu , Phy. Rev. Lett. 2020, 125, 267205. 10.1103/PhysRevLett.125.267205 33449751

[97] J. Xie , H. Qin , Y. Hao , B. Cheng , W. Liu , S. Ren , G. Zhou , Z. Ji , J. Hu , Sci. Rep. 2017, 7, 45642. 28393834 10.1038/srep45642 PMC5385869

[98] D. Afanasiev , J. R. Hortensius , M. Matthiesen , S. Manas‐Valero , M. Siskins , M. Lee , E. Lesne , H. S. J. van der Zant , P. G. Steeneken , B. A. Ivanov , E. Coronado , A. D. Caviglia , Sci. Adv. 2021, 7, eabf3096. 34078601 10.1126/sciadv.abf3096 PMC8172129

[99] J. He , S. Li , A. Bandyopadhyay , T. Frauenheim , Nano Lett. 2021, 21, 463003. 10.1021/acs.nanolett.1c00520 33749285

[100] B. Nafradi , P. Szirmai , M. Spina , A. Pisoni , X. Mettan , N. M. Nemes , L. Forro , E. Horvath , Proc. Natl. Acad. Sci. USA 2020, 117, 6417. 32152127 10.1073/pnas.1915370117 PMC7104399

[101] G. Zhou , T. Li , Y. Wu , P. Wang , K. Leng , C. Liu , Y. Shan , L. Liu , Adv. Opt. Mater. 2020, 8, 2000046.

[102] T. G. Park , B. K. Choi , J. Park , J. Kin , Y. J. Chang , F. Rotermund , ACS Nano 2021, 15, 7756. 33761743 10.1021/acsnano.1c01723

[103] X. Wang , C. Xiao , H. Park , J. Zhu , C. Wang , T. Taniguchi , K. Watanabe , J. Yan , D. Xiao , D. R. Gamelin , W. Yao , X. Xu , Nature 2022, 604, 468. 35444320 10.1038/s41586-022-04472-z

[104] C. Bao , P. Tang , D. Sun , S. Zhou , Nat. Rev. Phys 2022, 4, 33.

[105] X. Chen , H. Wang , H. Lie , C. Wang , G. Wei , C. Fang , H. Wang , C. Geng , S. Liu , P. Li , H. Yu , W. Zhao , J. Miao , Y. Li , L. Wang , T. Niem , J. Zhao , Z. Wu , Adv. Mater 2022, 34, 2106172. 10.1002/adma.202106172 34816497

[106] A. Kimel , A. Zvezdin , S. Sharma , S. Shallcross , N. de Sousa , A. Garcia‐Martin , G. Salvan , J. Hamrle , O. Stejskal , J. McCord , S. Tacchi , G. Carlotti , P. Gambardella , G. Salis , M. Munzenberg , M. Schultze , V. Temnov , I. V. Bychkov , L. N. Kotov , N. Maccaferri , D. Ignatyeva , V. Belotelov , C. Donnelly , A. Hierro Rodriguez , I. Matsuda , T. Ruchon , M. Fanciulli , M. Sacchi , C. R. Du , H. Wang , et al., J. Phys. D: Appl. Phys. 2022, 55, 463003.

[107] T. S. Lan , B. Ding , B. Liu , Nano Sel. 2020, 1, 298.

[108] M. S. Choi , N. Ali , T. D. Ngo , H. Choi , B. Oh , H. Yang , W. J. Yoo , Adv. Mater. 2022, 34, 2202408. 10.1002/adma.202202408 35594170

[109] K. Parto , A. Pal , T. Chavan , K. Agashiwala , C. H. Yeh , W. Cao , K. Banerjee , Phys. Rev. Appl. 2021, 15, 298.

[110] O. Thiabgoh , T. Eggers , M. H. Phan , Sens. Actuators, A 2017, 265, 120.

[111] V. Ortiz Jimenez , V. Kalappattil , T. Eggers , M. Bonilla , S. Kolekar , P. T. Huy , M. Batzill , M. H. Phan , Sci. Rep. 2020, 10, 4789. 32179867 10.1038/s41598-020-61798-2 PMC7075862

[112] M. H. Phan , H. X. Peng , Prog. Mater. Sci. 2008, 53, 323.

[113] V. Ortiz Jimenez , K. Y. Hwang , D. Nguyen , Y. Rahman , C. Albrecht , B. Senator , O. Thiabgoh , J. Devkota , V. D. A. Bui , D. S. Lam , T. Eggers , M. H. Phan , Biosensors 2020, 12, 517. 10.3390/bios12070517 PMC9313129 35884320

[114] F. X. Qin , H. X. Peng , M. H. Phan , L. V. Panina , M. Ipatov , V. Zhukova , A. Zhukov , J. Gonzalez , J. Appl. Phys. 2010, 107, 09A314.

[115] D. L. Duong , S. J. Yun , Y. Kim , S. G. Kim , Y. H. Lee , Appl. Phys. Lett. 2019, 115, 242406.

[116] B. Song , S. J. Yun , J. Jiang , J. Avila , K. Beach , W. Choi , Y. M. Kim , D. Yoon , H. Terrones , Y. J. Song , M. C. Asensio , D. L. Duong , Y. H. Lee , Phys. Rev. B. 2021, 103, 094432.

[117] J. Jiang , L. A. T. Nguyen , T. D. Nguyen , D. H. Luong , D. Y. Kim , Y. Jin , P. Kim , D. L. Duong , Y. H. Lee , Phys. Rev. B 2021, 103, 014441.

[118] D. T. X. Dang , R. K. Barik , M. H. Phan , L. M. Woods , J. Phys. Chem. Lett. 2022, 13, 8879. 36125200 10.1021/acs.jpclett.2c01925

[119] H. Munekata , T. Abe , S. Koshihara , A. Oiwa , M. Hirasawa , S. Katsumoto , Y. Iye , C. Urano , H. Takagi , J. Appl. Phys. 1997, 81, 4862.

[120] B. Marfoua , J. Hong , Phys. Chem. Chem. Phys. 2022, 24, 22523. 36107022 10.1039/d2cp03011j

[121] Q. Li , C. X. Zhang , D. Wang , K. Q. Chen , L. M. Tang , Mater. Adv. 2022, 3, 2927.

[122] J. Du , C. Xia , W. Xiong , Y. Lia , J. Li , Nanoscale 2017, 9, 17585. 29114682 10.1039/c7nr06473j

[123] C. Freysoldt , B. Grabowski , T. Hickel , J. Neugebauer , G. Kresse , A. Janotti , C. G. Van de Walle , Rev. Mod. Phys. 2014, 86, 253.

[124] J. Heyd , G. E. Scuseria , M. Ernzerhof , J. Chem. Phys. 2003, 118, 8207.

[125] G. Kresse , J. Furthmüller , Phys. Rev. B 1996, 54, 11169. 10.1103/physrevb.54.11169 9984901

[126] S. Grimme , S. Ehrlich , L. Goerigk , J. Comp. Chem. 2011, 32, 1456. 21370243 10.1002/jcc.21759

[127] C. Freysoldt , J. Neugebauer , Phys. Rev. B 2018, 97, 205425.

[128] N. L. Nair , E. Maniv , C. John , S. Doyle , J. Orenstein , J. G. Analytis , Nat. Mater. 2020, 19, 153. 31685945 10.1038/s41563-019-0518-x

[129] A. David , P. Rakyta , A. Kormanyos , G. Burkard , Phys. Rev. B 2019, 100, 085412.

[130] L. Sun , L. Rademaker , D. Maure , A. Scarfato , A. Pasztor , I. Gutierrez‐Lezama , Z. Wang , J. Martinez‐Castro , A. F. Morpurgo , C. Renner , Nat. Commun. 2023, 14, 3771. 37355633 10.1038/s41467-023-39453-x PMC10290717

[131] S. Shrestha , M. Cotlet , ACS Appl. Opt. Mater. 2023, 1, 1192.

[132] Y. Yoon , Z. Zhang , R. Qi , A. Y. Joe , R. Sailus , K. Watanabe , T. Taniguchi , S. Tongay , F. Wang , Nano. Lett. 2022, 22, 10140. 36485010 10.1021/acs.nanolett.2c04030

[133] N. Sokolowski , S. Palai , M. Dyksik , K. Posmyk , M. Baranowski , A. Surrente , D. Maude , F. Carrascoso , O. Cakiroglu , E. Sanchez , A. Schubert , C. Munuera , T. Taniguchi , K. Watanabe , J. Hagel , S. Brem , A. Castellanos‐Gomez , E. Malic , P. Plochoka , 2D Mater. 2023, 10, 034003.

[134] M. Z. Bellus , M. Li , S. D. Lane , F. Ceballos , Q. Cui , X. C. Zeng , H. Zhao , Nanoscale Horiz. 2017, 2, 31. 32260674 10.1039/c6nh00144k

[135] A. Chaves , J. G. Azadani , H. Alsalman , D. R. da Costa , R. Frisenda , A. J. Chaves , S. H. Song , Y. D. Kim , D. He , J. Zhou , A. Castellanos‐Gomez , F. M. Peeters , Z. Liu , C. L. Hinkle , S. H. Oh , P. D. Ye , S. J. Koester , Y. H. Lee , P. Avouris , X. Wang , T. Low , npJ 2D Mater. Appl. 2020, 4, 29.

[136] L. Du , T. Hasan , A. Castellanos‐Gomez , G. B. Liu , Y. Yao , C. N. Lau , Z. Sun , Nat. Rev. Phys. 2021, 3, 193.

[137] D. Zhong , K. L. Seyler , X. Lingpeng , N. P. Wilson , T. Taniguchi , K. Watanabe , M. A. McGuire , K. M. C. Fu , D. Xiao , W. Yao , X. Xu , Nat. Nano. 2020, 15, 187. 10.1038/s41565-019-0629-1 31988503

[138] E. M. Choi , K. I. Sim , K. S. Burch , Y. H. Lee , Adv. Sci. 2022, 9, 2200186. 10.1002/advs.202200186 PMC9313546 35596612

[139] D. Zhong , K. L. Seyler , X. Linpeng , R. Cheng , N. Sivadas , B. Huang , E. Schmidgall , T. Taniguchi , K. Watanabe , M. A. McGuire , W. Yao , D. Xiao , K. M. C. Fu , X. Xu , Sci. Adv. 2017, 3, 120. 10.1126/sciadv.1603113 PMC5451195 28580423

[140] D. L. Duong , S. J. Yun , Y. H. Lee , ACS Nano 2017, 11, 11803. 29219304 10.1021/acsnano.7b07436

[141] T. S. Ghiasi , A. A. Kaverzin , P. J. Blah , B. J. van Wees , Nano Lett. 2019, 19, 5959. 31408607 10.1021/acs.nanolett.9b01611 PMC6746057

[142] B. Zhao , B. Karpiak , D. Khokhriakov , A. Johansson , A. M. Hoque , X. Xu , Y. Jiang , I. Mertig , S. P. Dash , Adv. Mater. 2020, 32, 2000818. 10.1002/adma.202000818 32776352

[143] K. Uchida , S. Takahashi , K. Harii , J. Ieda , W. Koshibae , K. Ando , S. Maekawa , E. Saitoh , Nature 2008, 455, 778. 18843364 10.1038/nature07321

[144] K. Uchida , Proc. Japan Acad. Ser. B. 2021, 97, 69. 33563879 10.2183/pjab.97.004 PMC7897901

[145] S. K. Lee , W. Y. Lee , T. Kikkawa , C. T. Le , M. S. Kang , G. S. Kim , A. D. Nguyen , Y. S. Kim , N. W. Park , E. Saitoh , Adv. Funct. Mater. 2020, 30, 2003192.

[146] J. R. Schaibley , H. Yu , G. Clark , P. Rivera , J. S. Ross , K. L. Seyler , W. Yao , X. Xu , Nat. Rev. Mater. 2016, 1, 16055.

[147] H. Yu , W. Yao , Nat. Mater. 2017, 16, 876. 28850121 10.1038/nmat4979

[148] G. Aivazian , Z. Gong , A. M. Jones , R.‐L. Chu , J. Yan , D. G. Mandrus , C. Zhang , D. Cobden , W. Yao , X. Xu , Nat. Phys. 2015, 11, 148.

[149] T. Norden , C. Zhao , P. Zhang , R. Sabirianov , A. Petrou , H. Zeng , Nat. Commun. 2019, 10, 4163. 31519871 10.1038/s41467-019-11966-4 PMC6744439

[150] J. He , S. Li , L. Zhou , T. Fraenheim , J. Phys. Chem. Lett. 2022, 13, 2765. 35315669 10.1021/acs.jpclett.2c00443

[151] A. V. Kimel , M. Li , Nat. Rev. Mater. 2019, 4, 189.

[152] Y. Li , M. Koshino , Phys. Rev. B 2019, 99, 075438.

[153] S. Lee , D. J. P. de Sousa , Y. K. Kwon , F. de Juan , Z. Chi , F. Casanova , T. Low , Phys. Rev. B 2022, 106, 165420.

[154] N. Ontoso , C. K. Safeer , F. Herling , J. Ingla‐Aynés , H. Yang , Z. Chi , B. Martin‐Garcia , I. Robredo , M. G. Vergniory , F. de Juan , M. Reyes Calvo , L. E. Hueso , F. Casanova , Phys. Rev. Appl. 2023, 19, 014053.

[155] J. X. Lin , Y. H. Zhang , E. Morissette , Z. Wang , Science 2022, 375, 437. 34990215 10.1126/science.abh2889

[156] H. Yang , L. Liu , X. Yang , X. Wu , Y. Huang , H. J. Gao , Y. Wang , Nano Res. 2023, 16, 2579.

[157] X. Sui , T. Hu , J. Wang , B. L. Gu , W. Duan , M. S. Miao , Phys. Rev. B 2017, 96, 041410.

[158] X. Liu , A. P. Pyatakov , W. Ren , Phys. Rev. Lett. 2020, 125, 247601. 33412016 10.1103/PhysRevLett.125.247601

[159] H. Bouzid , S. Rodan , K. Singh , Y. Jin , J. Jiang , D. Yoon , H. Y. Song , Y. H. Lee , APL Mater. 2021, 9, 323.

[160] B. Li , B. Li , T. Xing , N. Zhong , L. Huang , N. Lei , J. Zhang , J. Li , Z. Wei , Nat. Commun. 2017, 8, 1958. 29208966 10.1038/s41467-017-02077-z PMC5717146

[161] H. C. Bouzid , R. Sahoo , S. J. Yun , K. Singh , Y. Jin , J. Jiang , D. Yoon , H. Y. Song , G. Kim , W. Choi , Y. M. Kim , Y. H. Lee , Adv. Funct. Mater. 2021, 31, 2102560.

